# A systematic review of economic evidence for community‐based obesity prevention interventions in children

**DOI:** 10.1111/obr.13592

**Published:** 2023-06-12

**Authors:** Marufa Sultana, Melanie Nichols, Marj Moodie, Steven Allender, Vicki Brown

**Affiliations:** ^1^ Deakin Health Economics, Institute for Health Transformation, School of Health and Social Development Deakin University Geelong Victoria Australia; ^2^ Global Centre for Preventive Health and Nutrition (GLOBE), Institute for Health Transformation, School of Health and Social Development Deakin University Geelong Victoria Australia

**Keywords:** community, cost, obesity, prevention

## Abstract

Multicomponent community‐based obesity prevention interventions that engage multiple sectors have shown promise in preventing obesity in childhood; however, economic evaluations of such interventions are limited. This systematic review explores the methods used and summarizes current evidence of costs and cost‐effectiveness of complex obesity prevention interventions. A systematic search was conducted using 12 academic databases and grey literature from 2006 to April 2022. Studies were included if they reported methods of costing and/or economic evaluation of multicomponent, multisectoral, and community‐wide obesity prevention interventions. Results were reported narratively based on the Consolidated Health Economic Evaluation Reporting Standards. Seventeen studies were included, reporting costing or economic evaluation of 13 different interventions. Five interventions reported full economic evaluations, five interventions reported economic evaluation protocols, two interventions reported cost analysis, and one intervention reported a costing protocol. Five studies conducted cost‐utility analysis, three of which were cost‐effective. One study reported a cost‐saving return‐on‐investment ratio. The economic evidence for complex obesity prevention interventions is limited and therefore inconclusive. Challenges include accurate tracking of costs for interventions with multiple actors, and the limited incorporation of broader benefits into economic evaluation. Further methodological development is needed to find appropriate pragmatic methods to evaluate complex obesity prevention interventions.

**Abbreviations**
BMIz: body mass index *z* score
CBA: cost–benefit analysis
CBIs: community‐based interventions
CEA: cost‐effectiveness analysis
CHEERS: Consolidated Health Economic Evaluation Reporting Standards
CUA: cost‐utility analysis
DALY: disability‐adjusted life year
HALY: health‐adjusted life year
HRQoL: health‐related quality of life
HTA: Health Technology Analysts
ICER: incremental cost‐effectiveness ratio
PRISMA: Preferred Reporting Items for Systematic Reviews and Meta‐Analyses
QALY: quality‐adjusted life year
ROI: return on investment
USD: United States dollar

## 1 INTRODUCTION

Childhood overweight and obesity are among the major public health challenges of the 21st century.^1^ The prevalence of children living with overweight and obesity is high globally, estimated at over 340 million children and adolescents aged 5–19 years and approximately 39 million children aged under 5 years.^1^ Overweight and obesity in childhood and adolescence are associated with lower health‐related quality of life (HRQoL) and additional healthcare costs.^2^, ^3^, ^4^, ^5^, ^6^ Obesity in childhood and adolescence substantially increases the risk of chronic disease later in life, including type 2 diabetes mellitus, cardiovascular disease, hypertension, and various types of cancers.^1^ The high health and economic burden of obesity^7^, ^8^ clearly demonstrates the need for childhood obesity prevention interventions that are effective, scalable, and economically viable in terms of cost‐effectiveness.^9^, ^10^

Tackling the issue of childhood obesity is challenging, given the wide range of complex, multifactorial determinants across multiple ecological levels.^11^ Multisetting, multistrategy interventions that incorporate community engagement and systems change (community‐based interventions [CBIs]) have been identified as promising in reducing the prevalence of obesity at the population level. This in contrast with single‐sector interventions, which cannot address the multifactorial root causes of obesity in a meaningful way. For instance, Shape‐Up Somerville was a community‐based environmental change intervention across households, schools, and communities conducted in Massachusetts, USA, that demonstrated reductions of body mass index *z* score (BMIz) among school‐aged children.^12^ Similarly, the Romp & Chomp intervention conducted in Victoria, Australia, targeted children aged from birth to 5 years through community capacity building and environmental changes in early childhood education and care settings and demonstrated a significant difference in BMI among children aged 3.5 years between the intervention and comparator groups.^13^, ^14^

Given scarce resources, such CBIs must be both effective and cost‐effective, and economic evaluation of multisetting, multicomponent CBIs is important to inform decision‐makers on which interventions represent good value for money.^9^, ^15^ However, the economic evaluation of complex interventions for obesity prevention presents several methodological challenges.^10^ These challenges include limited evidence that interventions incorporating changes in behavior, settings, and environments result in a significant outcome, such as reduced BMI, within relatively limited study timeframes; and the complexity of rigorously attributing costs and effects across interventions involving multiple players at multiple levels of the system simultaneously.^10^

Whilst systematic reviews of the economic evidence for obesity prevention interventions have been published,^15^, ^16^ to the best of our knowledge, there are no current systematic reviews of the economic evidence for multicomponent and multisectoral community‐based interventions targeting obesity prevention. A broad 2019 systematic review of economic evaluations of obesity interventions in children and adolescents^16^ included 28 prevention interventions.^16^ The search strategy used did not however include terms specific to CBIs, as the aim of the paper was to summarize the economic evidence for childhood obesity prevention and treatment interventions more generally and to assess the economic methods applied. As a result, the review did not provide a comprehensive overview of the state of the economic evidence for complex CBIs for childhood obesity prevention specifically, and in fact only included economic evaluations of two CBIs^17^, ^18^ at that time. In 2014, Flego et al. conducted a narrative review to synthesize the evidence of the cost‐effectiveness of CBIs for obesity prevention.^15^ The review included 11 studies comprising full economic evaluations^17^, ^18^, ^19^, ^20^, ^21^, ^22^, ^23^, ^24^ and did not include partial economic evaluation (e.g., cost analysis) or protocol papers. This limits the evidence that could be synthesized on the cost of intervention, particularly if interventions may not have had a statistically significant effect on their primary outcome and so a full economic evaluation may never have been published. This also limits the evidence for contemporaneous methods to undertaken economic evaluations of CBIs, and this may slow the development of robust and rigorous methods for estimating the value for money of these complex interventions. In addition, because cost‐effectiveness research in this area was in its infancy at the time of this review,^15^ the definition of CBIs used was also broad. This resulted in the inclusion of seven studies that were predominantly conducted in the school setting, with minimal intervention engagement from other settings (e.g., households and community).^19^, ^20^, ^21^, ^22^ More recent developments in CBIs include an increasing focus on meaningfully engaging multiple community sectors using a participatory approach.^25^ This is distinct from earlier studies, which may have focused on a single sector such as a school, with relatively minimal engagement or participation from the wider community.^25^ Given the advances in CBI research made in the years since the review was published, it is likely that only four of the included economic evaluation studies would now be considered as evaluating multisectoral CBIs (rather than school‐based interventions). Therefore, a significant gap in synthesizing the economic evidence for CBIs exists, particularly when considering the increasing interest in CBIs and the new studies conducted within the last decade. To address these gaps, it is necessary to synthesize evidence by including a broader range of economic studies that investigate community‐based interventions for obesity prevention involving multiple stakeholders. As such, the objective of this review is to synthesize up‐to‐date evidence of the costs and cost‐effectiveness of community‐based obesity prevention interventions and to provide a comprehensive examination of the methods used in published studies. This analysis will generate evidence of the economic credentials of CBIs for use in resource allocation and decision‐making and underpin discussion on the future research directions required to overcome the significant challenges in understanding the costs and benefits of multicomponent and multisectoral obesity prevention interventions.

## 2 METHODS

The systematic review was registered with PROSPERO (CRD42021262817) and followed the Preferred Reporting Items for Systematic Reviews and Meta‐Analyses (PRISMA) guidelines.^26^ The PRISMA Checklist is in Table S1.

### 2.1 Search strategies and screening procedures

A systematic electronic search was conducted through April 2022 of 12 academic databases (Academic Search Complete, CINAHL Complete, EconLit, Global Health, MEDLINE Complete, APA PsycINFO, SPORTDiscus, Scopus, Embase, PubMed, Health Technology Assessment Database, and Cochrane Library) and the grey literature. The search strategy was developed in conjunction with a subject‐specific librarian. Relevant grey literature was identified through multiple Google searches, including both a general and targeted search. General search terms were based on those used in the academic database search, with screening of the first 10 pages of search hits. General search terms were then combined with the names of identified CBIs from a recent systematic review^25^ to conduct a targeted grey literature search. Full details of the search strategies are presented in Table S2.

Study inclusion criteria were as follows:

- Intervention in the general child population (aged from birth to 18 years);
- Multicomponent and multisetting community‐based obesity prevention intervention, defined as targeting a whole community and engaging at least two community sectors (e.g., households/families, schools, media, businesses, health services, community/recreation centers, and local governments) through a participatory approach, focused on the prevention of childhood obesity^25^;
- Reported either the protocol for or the results from a full economic evaluation (cost‐effectiveness analysis [CEA], cost–utility analysis [CUA], cost–benefit analysis [CBA]) or a costing study, of an obesity prevention CBI;
- Published between January 2006 and April 2022. The timeframe was started at 2006 for the relative recency of most CBIs for obesity prevention^25^, ^27^, ^28^ and the aim of providing a synthesis of up‐to‐date economic evidence;
- Published in the English language;
- Original articles, study protocols, reports. Protocol papers were included in the review to include the most recent methodologies employed, although economic results may not be available for these interventions.

Studies were excluded if interventions were single‐component, not community‐based, engaged only a single sector, targeted high‐risk individuals or specific population groups, or focused on the treatment of obesity.

Identified studies were initially imported into EndNote, and duplicates were removed. The remaining records were exported to Covidence (https://www.covidence.org) for independent title and abstract screening by two reviewers (MS, VB). Full text review was undertaken, with all conflicts resolved by consensus of both reviewers. Reference lists of all included studies were hand‐searched for additional inclusions by one reviewer (MS).

### 2.2 Data extraction and synthesis

A data extraction template was developed in Microsoft Excel following the Consolidated Health Economic Evaluation Reporting Standards (CHEERS) checklist.^29^ Extracted information for each study included study design and settings, target population, sample size, brief summary of intervention and comparator, study perspective, discount rate, time horizon, measurement of effectiveness, choice of health outcomes, measurement and valuation of preference based outcomes, resource use and cost categories, currency, reference year, model assumptions, characterization of uncertainty and heterogeneity, sensitivity analysis, results, limitations, source of funding, and conflicts of interest. Data extraction was completed by one reviewer (MS) and checked by another reviewer (VB) to ensure all relevant information was extracted.

Findings were narratively synthesized, with a detailed narrative summary of the relevant CHEERS items^29^ for each included study available in Table S3. Reported costs of each included intervention were converted to 2022 US dollars (USD) to facilitate discussion of the major findings. Conversion was carried out using the EPPI‐CCEMG cost calculator (https://eppi.ioe.ac.uk/costconversion/).

## 3 RESULTS

### 3.1 Study inclusion

**FIGURE 1:**
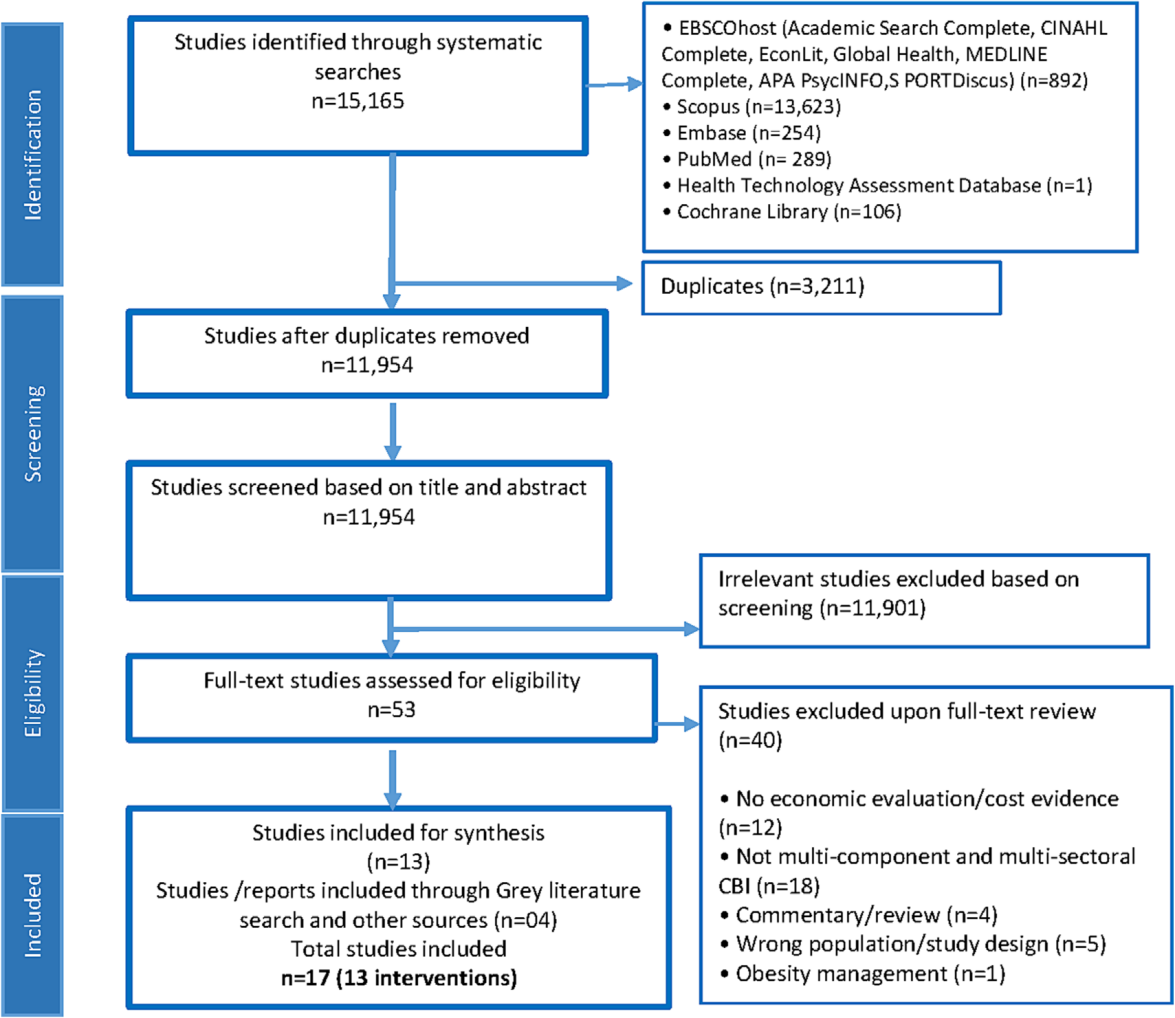
PRISMA flow chart of study selection on economic evidence of obesity prevention intervention.

The systematic search identified 15,165 records published between January 2006 and April 2022 (Figure 1). After removal of duplicates (*n* = 3211), title and abstract screening excluded 11,901 studies. Full‐text screening of the remaining 53 studies excluded a further 40 studies, leaving 13 studies that were included from the academic database search. Four further studies^24^, ^30^, ^31^, ^32^ were identified through the grey literature search, resulting in a total of 17 studies included in this review.^17^, ^18^, ^24^, ^30^, ^31^, ^32^, ^33^, ^34^, ^35^, ^36^, ^37^, ^38^, ^39^, ^40^, ^41^, ^42^, ^43^

From the 17 included studies, 15 discrete economic evaluations, protocols, or costing studies of 13 CBIs were identified (Table 1). The economic evaluation of a CBI was reported in full in the publication by Ananthapavan et al.^34^ and also included as part of a larger priority‐setting study.^35^ The protocol for economic evaluation of Whole of Systems Trial of Prevention Strategies (WHO STOPS) for Childhood Obesity was reported in detail in the publication by Sweeney et al.^39^ and a brief summary included in the wider study protocol.^33^ Likewise, the protocol for the economic evaluation of Obesity Prevention in Communities (OPIC) was reported in two studies.^40^, ^41^ Conversely, one report included the economic evaluation of two different CBIs (Be Active Eat Well [BAEW] and A Pilot Program for Lifestyle and Exercise [APPLE]).^24^

**TABLE 1:** Summary of included studies (*n* = 17 studies incorporating 15 discrete economic evaluations, protocols or costing studies of 13 CBIs).

Name of intervention (*n* = 13)	Country	Full EE				Protocol for full EE			Cost analysis	
		CEA	CUA	CEA & CUA	CBA/ROI	CEA	CUA	CEA & CUA	Protocol	With results
A Pilot Program for Lifestyle and Exercise (APPLE)^17^, ^24^	New Zealand	1	1							
Be Active, Eat Well (BAEW)^18^, ^24^	Australia			1						
	New Zealand		1							
CBI (hypothetical)^34^, ^35^	Australia		1^a^							
Childhood Obesity Research Demonstration (CORD)^32^	USA					1^b^				
Fun 'n Healthy in Moreland!^43^	Australia									1
Health, Exercise, Nutrition for the Really Young (HENRY)^36^	United Kingdom					1				
Healthy Habits, Happy Homes (4H)^38^	Scotland								1	
Obesity Prevention and Lifestyle (OPAL)^30^	Australia									1
Obesity Prevention In Communities (OPIC)^40^, ^41^	Fiji, Tonga, New Zealand, Australia						1^a^			
Optimising Family Engagement in HENRY (OFTEN)^37^	United Kingdom					1				
Romp & Chomp (R&C)^42^	Australia			1						
Shape Up Somerville (SUS)^31^	USA				1					
WHO STOPS Childhood Obesity (WHO STOPS)^33^, ^39^	Australia							1^a^		
Number of discrete economic analyses (*n* = 15) from 17 included studies		1	3	2	1	3	1	1	1	2

Abbreviations: CBA, cost benefit analysis; CBI, community‐based intervention; CEA, cost effectiveness analysis; CUA, cost utility analysis; EE, economic evaluation; ROI, Return on investment; USA, United States of America.

^a^ The same analysis was reported in multiple studies.

^b^ Type of economic evaluation was not clearly stated.

### 3.2 Summary of intervention characteristics

Twelve CBIs were conducted exclusively in high‐income countries (HICs) (Table 1).^17^, ^18^, ^24^, ^30^, ^31^, ^32^, ^33^, ^34^, ^35^, ^36^, ^37^, ^38^, ^39^, ^42^, ^43^ The OPIC study was conducted in both high‐income and upper‐middle‐income countries from the Western Pacific region (Fiji, Tonga, New Zealand, Australia).^40^, ^41^ Six CBIs were implemented in Australia (BAEW, Fun 'n Healthy in Moreland!, hypothetical CBI, Obesity Prevention and Lifestyle [OPAL], Romp & Chomp [R&C], WHO STOPS),^18^, ^30^, ^33^, ^34^, ^35^, ^39^, ^42^, ^43^ two in the United States (Childhood Obesity Research Demonstration [CORD], Shape Up Somerville [SUS]),^31^, ^32^ two in the United Kingdom (Health, Exercise, Nutrition for the Really Young [HENRY] and Optimising Family Engagement in HENRY [OFTEN]),^36^, ^37^ one in New Zealand (APPLE),^17^ and one in Scotland (Healthy Habits, Happy Homes [4H])^18^ (Table 1). One economic evaluation applied the intervention cost and effect data from an intervention in the Australian setting (BAEW), to estimate the cost‐effectiveness if it were implemented in the New Zealand population.^24^ Two CBIs (4H, R&C) targeted early childhood specifically (approximately 0–5 years),^38^, ^42^ six CBIs (APPLE, BAEW, Fun 'n Healthy in Moreland!, OPAL, SUS, WHO STOPS)^17^, ^18^, ^24^, ^30^, ^31^, ^32^, ^33^, ^39^, ^40^, ^41^, ^43^ targeted primary (elementary) school aged children (ages approximately 4–13 years) and one CBI (OPIC)^40^, ^41^ targeted adolescents (aged 12–18 years). Ananthapavan et al. estimated the effectiveness of complex CBIs for obesity prevention through literature review and meta‐analysis and designed a hypothetical CBI targeting Australian children aged 5–18 years based on the literature to model cost‐effectiveness.^34^, ^35^

The majority of interventions (APPLE, BAEW, Fun 'n Healthy in Moreland!, HENRY, HENRY‐OFTEN, OPAL, SUS, OPIC) were undertaken in school settings, with other sectoral engagement including early education and care, local health services, community and support organizations, households, and restaurants.^17^, ^18^, ^30^, ^31^, ^36^, ^37^, ^40^, ^41^, ^43^ BAEW, for example, targeted the whole community through engaging local health agencies, community stakeholders, and primary schools.^18^ The CORD CBI primarily focused on primary healthcare clinics along with other sectors, including education (e.g., education centers and schools).^32^ The R&C intervention was a community‐based and community‐wide obesity prevention intervention, incorporating strong engagement with early education and care settings.^42^ CBIs evaluated by Ananthapavan et al.,^34^, ^35^ and the WHO STOPS intervention^33^, ^39^ utilized a systems approach through engaging a range of organizations and community settings (e.g., partnerships between researchers and community leaders, parents and community leaders from local government, health agencies, businesses, and clubs).

### 3.3 Summary of full economic evaluations (*n* = 7)

Seven full economic evaluations of five different CBIs were identified^17^, ^18^, ^24^, ^31^, ^34^, ^35^, ^42^ (Table 2). One full economic evaluation was conducted alongside the research trial,^17^ whereas three full economic evaluations used best available evidence to conduct modeled economic evaluations.^24^, ^34^, ^35^, ^42^ Two studies conducted both within‐trial and modeled economic evaluation,^18^, ^42^ and one study utilized a return on investment (ROI) ratio to report full economic evaluation.^31^ The majority of the full economic evaluations (*n* = 4) adopted a societal perspective,^17^, ^18^, ^31^, ^34^, ^35^ with three adopting a funder/health sector perspective (Table 2).^24^, ^42^ Comparators included “current practice”^18^, ^34^, ^35^ and “no intervention.”^17^, ^24^, ^31^, ^42^ The time horizon for trial‐based economic evaluations (*n* = 3) ranged from 3 to 10 years.^17^, ^18^, ^31^ The time horizon for modeled CUAs ranged from 10 years to lifetime (up to 100 years of age).^18^, ^24^, ^34^, ^35^, ^42^ The discount rate used either for costs or costs and benefits was reported as 3%,^18^, ^31^, ^34^, ^35^ 3.5%,^24^ or 5%^17^, ^42^ (Table 2).

**TABLE 2:** Brief summary of the cost and cost‐effectiveness methods of included studies (costs adjusted to USD 2022 price year [in brackets]).

Author, country, currency & price	Type of EE, perspective	Target population	Intervention	Major cost categories	Summary of EE results
**Full economic evaluations**					
McAuley et al.^17^ New Zealand NZD2006	CEA, Societal	NZ children aged 5–12 years	A Pilot Program for Lifestyle and Exercise (The APPLE project): Nutrition‐based interventions with PA provided through Community Activity Coordinators (ACs) at school.	‐Travel ‐Time ‐Materials ‐Overheads	Aged 7 years: NZD854 (USD810) per kilogram of weight‐gain prevented per year Aged 13 years: NZD332 (USD314) per kilogram of weight‐gain prevented per year
Health Technology Analysts.^24^ New Zealand NZD2010	CUA, Healthcare/funder	NZ children aged 9 years; general population		‐Administrative ‐Time ‐Equipment ‐Materials ‐Overheads ‐Healthcare costs for different health states	ICER: NZD 205,101 (USD 173,062) per QALY gained *Not cost‐effective*
Moodie et al.^18^ Australia AUD2006	CUA, Societal	Children aged 4–12 years	Be Active Eat Well (BAEW): Behavior change whole‐of‐community intervention.	‐Travel ‐Time ‐Materials	Trial: Cost per DALY saved of AUD29,798 (USD 29,575) Modeled: Cost per DALY saved: AUD 20,227 (USD20,076) *Cost‐effective*
Health Technology Analysts^24^ New Zealand NZD2010	CUA, Healthcare/funder	NZ children aged 8 years; general, Māori and Pacific populations		Overall cost per participant, no separate cost categories	ICER: NZD168,391 (USD142,087) per QALY gained (General population) ICER: NZD123,536 (USD104,239) per QALY gained (Māori population) ICER: NZD154,178 (USD130,093) per QALY gained (Pacific population) *N*o*t cost‐effective*
Ananthapavan et al.^34^ Australia AUD2010 Also reported in: Ananthapavan et al.^35^	CUA, Limited societal	Australian children 5–18 years	A hypothetical CBI was designed for Australian setting based on available literature and subsequent meta‐analysis.	‐Time ‐Healthcare saving costs from averted diseases	ICER: AUD8,155 (USD8,893) per HALY gained *Cost‐effective*
Tran et al.^42^ Australia AUD2018	CEA & CUA, Funder	Australian children aged 0–5 years	Romp & Chomp intervention was implemented in Geelong and Queenscliff cities of Victoria that promoted healthy eating and active play.	‐Travel ‐Time ‐Intervention materials ‐Healthcare cost‐savings	ICER: AUD1,126 (USD896) per BMI unit avoided ICER: AUD20,574 (USD15,488) per QALY gained *Cost‐effective*
Coffield et al.^31^ USA USD2014	ROI, Modified societal	Children grades 1–3	Shape Up Somerville (SUS): Influence multiple aspects at schools, home environment and range of community initiatives.	‐Labor ‐Capital equipment ‐Materials ‐Facility cost ‐Productivity	‐Estimated averted costs: USD500,000 (USD576,278) ‐Estimated benefits: USD197,120 (USD227,192) ‐Projected $1.51 (USD1.75) in savings for every $1.00 invested in the program ‐ROI estimates show benefits exceed costs.
**Cost analyses**					
Waters et al.^43^ Australia AUD2019	Cost analysis NS	Children aged 4–13 years	Fun 'n healthy in Moreland!: Nutrition based interventions together with physical activity implemented through school and community to prevent obesity.	‐Time ‐School resources ‐Parent expenses	‐Cost per student AUD229 (USD168) ‐Family cost per child per year AUD65 (USD48)
Bell et al.^30^ Australia AUD2016	Cost analysis NS	Children aged 9–11 years	Obesity Prevention and Lifestyle (OPAL): healthy eating and physical activity in each community through range of themes.	‐Coordination ‐Administration ‐Marketing ‐Staff salaries ‐Training	Average cost per child was AUD288 (USD229)
**Economic evaluation protocols**					
O'Connor et al.^32^ USA USD2014	CEA protocol, NS	Children aged 2–12 years	Childhood Obesity Research Demonstration (CORD): healthy eating and PA together with community‐level programs.	‐Labor ‐In‐kind cost ‐Project expenses	NA
Bryant et al.^36^ UK Currency not stated	CEA protocol, NS	Parents of preschool children	HENRY (Health, Exercise, Nutrition for the Really Young): an 8‐week program designed to train center level and practitioner level trainings to provide parents with knowledge to support healthy lifestyle.	Parent expenses ‐Travel ‐Extra food expenses ‐Extra activities ‐Lost productivity	NA
Bryant et al.,^37^ UK Currency not stated	CEA protocol, NS	Parents of the selected children's centers	The Optimising Family Engagement in HENRY (OFTEN) trial: Health, Exercise, Nutrition program to support a healthy lifestyle in preschool children and their families.	‐Expenses of each of the additional activities to deliver the intervention	NA
Swinburn et al.^41^; Fiji, Tonga, New Zealand, Australia Local currencies2011 Also reported in: Swinburn et al.^40^	CUA protocol, NS	Adolescents aged 12–18 years	Obesity Prevention In Communities (OPIC): build community capacity to promote healthy eating and physical activity.	‐Costs of delivery of intervention ‐Healthcare costs associated with the chronic diseases considered	NA
Sweeney et al.^39^ Australia AUD2018 Also reported in: Allender et al.^33^	CUA protocol, Funder and societal	Children aged 8–12 years	WHO STOPS Childhood Obesity: collective approach including fostering, supporting and monitoring community engagement and community led actions to prevent obesity.	‐Time ‐Training ‐Equipment ‐Infrastructure ‐Communication and meeting ‐Venue rent	NA
**Costing protocols**					
Gillespie et al.,^38^ Scotland Currency not stated	Costing protocol. NS	Children aged 2–5.5 years	Healthy Habits Happy Homes (4H): Lifestyle change family‐based intervention	‐Time ‐Training ‐Travel ‐Delivery of interventions	NA

Abbreviations: AUD, Australian dollars; CEA, cost‐effectiveness analysis; CUA, cost‐utility analysis; DALY, disability adjusted life year; HALY, health‐adjusted life years; ICER, incremental cost‐effectiveness ratio; LGA, local government areas; NA, not applicable; NS, not stated; NZ, New Zealand; NZD, New Zealand dollar; QALY, quality‐adjusted life‐year; ROI, return on investment; USD, United States dollars.

The APPLE intervention has been the subject of two full economic evaluations.^17^, ^24^ The study by McAuley et al.^17^ conducted a within‐trial CEA from a societal perspective, providing evidence of cost per kilogram of weight gain prevented (additional cost of USD810 and USD314 per kilogram of weight‐gain prevented per year for children aged 7 and 13 years, respectively). This intervention was also the subject of a modeled CUA from a funder perspective, providing evidence of the cost per quality‐adjusted life‐year (QALY) gained for children aged 9 years in New Zealand.^24^ Utility weights were sourced from a comprehensive literature search and the evaluation used key input parameters based on the study by McAuley et al.^17^ Given the low incremental benefits from the intervention (0.007 QALYs gained), the modeled CUA was not cost‐effective, with an estimated cost per QALY gained of USD173,062.^24^

Similarly, the BAEW CBI has been the subject of two full economic evaluations.^18^, ^24^ The study by Moodie et al.^18^ conducted a modeled CUA of BAEW for the population of children aged 4–12 years in the Australian regional town of Colac (*n* = 2184). Costs and effects of BAEW were also extrapolated to the national level (assumed 10% of Australian primary school children).^44^, ^45^ Health benefits were measured as change in BMI and disability adjusted life years (DALYs) saved over the lifetime of the cohort using the ACE‐Obesity model,^45^ with an assumption of 100% maintenance of the intervention effect.^18^ DALYs averted were calculated as the difference in future outcomes between intervention and control groups, where impact of the change in BMI distribution on the incidence of nine diseases related to BMI was estimated using potential impact fractions. The intervention was estimated to be cost‐effective (cost per DALY saved Colac cohort: USD29,575; national cohort: USD20,075).

The BAEW CBI was also the subject of a modeled CUA where the effect of BAEW intervention was applied to the New Zealand general, Māori, and Pacific populations of children aged 9 years.^24^ The cost per participant applied to the model was calculated from the BAEW report.^46^ The time horizon for the evaluation was 92 years (lifetime), and the model assumed a 1% decay in intervention effect per annum after the fifth year of the intervention. No utility difference was applied to BMI categories for the base‐case analysis.^24^ In this analysis, BAEW was not cost‐effective (USD130,093; USD142,087; USD104,239 per QALY gained for Pacific, general and Māori populations within New Zealand, respectively).^24^ Both analyses revealed that interventions became not cost‐effective when incorporating intervention effect decay in sensitivity analyses (Tables 2 and S3).

Ananthapavan et al.^34^ reported the results of a modeled CUA of a hypothetical complex CBI targeting Australian children aged from 5 to 18 years. The analysis formed part of a national priority setting study^47^ and was conducted from a limited societal perspective. Costs were estimated using a variety of sources (e.g., trial data and the literature) and effectiveness was informed through a literature review and meta‐analysis. The analysis used the ACE‐Obesity Policy model^47^ to estimate health‐adjusted life years (HALYs) saved and healthcare cost‐savings from diseases averted as a result of intervention. The modeled intervention was cost‐effective, with an incremental cost effectiveness ratio (ICER) of USD9,077 per HALY gained. However, the intervention became not cost‐effective when the decay of intervention effect (5% per year) was considered in sensitivity analysis (Table S3). The threshold analysis indicated that the intervention effect would need to persist for a duration of 29 years for the intervention to be deemed cost‐effective.

Coffield et al. economically evaluated the SUS CBI that targeted children at elementary schools in Massachusetts.^31^ The economic benefits of this two‐year intervention were estimated through estimating an ROI. Costs were estimated using SUS project data and the intervention benefits for children and parents (healthcare cost saving and averted productivity losses) were projected over a 10‐year time horizon including depreciation of treatment effect using The estimated costs were USD576,278 with estimated net benefits of USD227,192. Results suggested a positive ROI, with USD1.75 savings for every USD1.00 invested in the program.^31^

The most recent full economic evaluation was a modeled CEA and CUA of the Romp & Chomp CBI if it were to be scaled up to target all Australian children aged 0–5 years.^42^ Intervention costs were estimated using a micro‐costing approach, and effect size was based on results from the repeat cross‐sectional quasi‐experimental evaluation of the project.^14^ The economic evaluation then used the Early Prevention of Obesity in CHildhood (EPOCH) model^48^ to predict individual level child BMI trajectories, weight status, and associated QALYs^2^ and healthcare costs from age 4 to 15 years as a result of intervention. The intervention was reported as cost‐effective, with an ICER of USD878 per BMI unit avoided and USD 15,488 per QALY gained.

### 3.4 Summary of protocols of full economic evaluations

Protocols for full economic evaluations of five CBIs, for which the results were not available, were included in this review.^32^, ^33^, ^36^, ^37^, ^39^, ^40^, ^41^ A detailed protocol for the economic evaluation of the WHO STOPS CBI was reported by Sweeney et al.,^39^ and briefly summarized in the study's main protocol paper.^33^ The economic evaluation as proposed aimed to undertake a CEA (cost per BMI unit saved) and a modeled CUA (cost per QALY gained) over the lifetime horizon. Data collection from various sources (e.g., project records and meeting minutes) was planned, across three intervention components: Community engagement and facilitation; backbone organization; and community‐led actions. HRQoL was planned to be assessed using the PedsQL^49^ instrument, and an existing multistate life table Markov model would estimate intervention cost‐effectiveness.^50^ Sensitivity analyses varying assumptions of intervention effect maintenance were planned.^39^ In addition, the dollar value of community resources mobilized for each $1 investment into community engagement and facilitation would also be calculated.

Two protocols for the OPIC intervention were published in 2007 and 2011,^40^, ^41^ which incorporated brief sections for the modeled economic evaluation. The resource use for intervention activities would be documented and estimated using trial records. Quality of life was to be measured at baseline and follow‐up, and the costs and outcome data would be combined with local data on obesity‐related diseases and their costs in order to estimate the disease burden and healthcare cost implications of changes in adolescent obesity. No further details on the proposed economic model were given.^40^, ^41^

Bryant et al. described a protocol to evaluate the feasibility of the HENRY intervention with an economic outcome of incremental cost per increase of parent engagement.^36^ This feasibility study aimed to determine the feasibility of recruiting local authorities, childcare centers, and parents to deliver the HENRY intervention. Aims also included examining acceptability of the trial to parents, childcare centers, and local authorities. Resource use information would be collected for healthcare utilizations, out‐of‐pocket payments, with productivity loss of parents (Table 2). Unit cost of resources would be sourced from the British National Formulary^51^ and the Personal Social Services Research Unit Costs of Health and Social Care.^52^

The protocol for HENRY (OFTEN) (HENRY with additional components) also aimed to evaluate parent engagement to implement intervention components (e.g., healthy diet and lifestyles for children).^37^ The proposed method included generating cost‐effectiveness acceptability curves to determine cost‐effectiveness of the intervention.^37^ A willingness‐to‐pay survey from a health commissioner perspective will be explored and used to inform the cost‐effectiveness thresholds for economic evaluation.^37^

The CORD CBI aimed to improve healthy eating and physical activity among children aged 2–12 years and to estimate the benefits that can be gained per dollar invested. The protocol outlined that activity‐based costing would be used to measure intervention cost longitudinally; however, additional details and methods for the economic evaluation were not clearly presented.^32^

### 3.5 Summary of cost analyses (*n* = 2) and protocols for cost‐analysis (*n* = 1)

Two cost analyses of CBIs were included in the review.^30^, ^43^ Waters et al. evaluated the cost of the Fun 'n Healthy in Moreland! CBI conducted in Australia, targeting children aged 4 to 13 years. The costs of intervention (e.g., time cost of community development workers, school resources, and parent expenditure) were apportioned equally between intervention schools to estimate the intervention cost per student. The estimated cost per student per year was USD168 per student per year for providers and USD48 for families.^43^

The evaluation of OPAL initially aimed to assess cost per QALY gained in a full economic evaluation. However, because of the repeat cross‐sectional evaluation design, quality of life changes among individuals were not estimated and so an economic evaluation was not undertaken. The costs of OPAL were calculated (e.g., development and administration and council expenditure) and divided by the number of individual children in the target age group in each of the intervention communities. The estimated cost per beneficiary was USD229 (67,322 beneficiaries).^30^

One protocol for a cost analysis was included in the review.^38^ The 4H CBI is being implemented in Scotland targeting early childhood to improve healthy lifestyle. Main cost categories include administration, travel, and training cost. However, no specific description of method in terms of perspective, time horizon, and discount rate (if applicable) was provided.^38^

## 4 DISCUSSION

This review narratively synthesized the economic evidence for multicomponent and multisectoral childhood obesity prevention CBIs. Of the five CUAs that have been published to date, three have been estimated as cost‐effective^18^, ^42^, ^47^ and two have been estimated as not cost‐effective.^24^ Three studies estimated CEA using different outcome measures^17^, ^42^ and were therefore not comparable. Five protocols for economic evaluations and one protocol for a costing study were also included in our review, highlighting the interest in better establishing the evidence for the cost and cost‐effectiveness of complex CBIs for obesity prevention. However, the relatively long period of time since publication of some of these protocols (for instance, the publications by O'Connor et al.^32^ and Swinburn et al.^40^, ^41^) suggest that the results from some analyses may not be made publicly available. While previous reviews have synthesized the economic evidence for obesity prevention interventions more generally,^16^ or for CBIs specifically,^15^ our review is stand‐alone and sufficiently different in terms of aims, the types of interventions considered, the type of economic evidence included, and the search strategies and methodology used. The studies by McAuley et al. (evaluating APPLE)^17^ and Moodie et al. (evaluating BAEW)^18^ were included in previous reviews by Flego et al.^15^ and Zanganeh et al.,^16^ and Flego's review also incorporated modeled economic evaluations for the APPLE and BAEW interventions from the grey literature.^24^ Since then however, there has been a significant increase in the economic evidence for multicomponent and multisectoral CBIs, with a further four full economic evaluations (CORD,^32^ a hypothetical CBI,^34^, ^35^ Romp & Chomp,^42^ and SUS^31^; representing a doubling of the cost‐effectiveness evidence since the time of the Flego et al. review)^15^ and two partial economic evaluations (Fun 'n healthy in Moreland!^43^ and OPAL^30^) published. Among them, three were new full economic evaluations of interventions that provide potentially more contemporaneous cost‐effectiveness evidence on which to base funding decisions (CORD,^32^ SUS,^31^ and hypothetical CBI^34^). Two of the included full economic evaluations were model based studies (hypothetical CBI^34^ and Romp & Chomp^42^) that utilized different time horizons and therefore provide contemporaneous evidence of the potential for longer term health benefits and healthcare cost‐savings from CBIs. In addition, our review includes partial economic evaluations (i.e., cost evidence) of two interventions.^30^, ^43^ And while the five protocols included in our review do not provide evidence of cost‐effectiveness, they do provide the reader with an overview of current methods for evaluation being used in the field.

Undertaking the economic evaluation of a CBI for obesity prevention is a complex task. The nature of complex CBIs means that more traditional study designs such as randomized controlled trials, with randomization at the individual participant level, may not be feasible. Study designs of the CBIs for which we identified economic evidence within our review included quasi‐experimental, cluster randomized controlled trials, longitudinal or repeated cross‐sectional studies. This makes the economic evaluation of these interventions challenging, given the inherent difficulties of measuring cost and effect where the repeat measurement of the same individual may not be possible. For example, Bell et al.^30^ reported that measurement of HRQoL changes was not practical for the OPAL intervention and economic evaluation was therefore not feasible, and a cost‐analysis was conducted instead. In addition, the nature of complex CBIs means that several, concurrent and interacting policy and practice interventions may be in place at the same time, limiting investigators' ability to separately identify and rigorously attribute intervention effects.^53^ Longitudinal studies could be a better solution to mitigate these challenges in conducting economic evaluations for such CBIs for obesity prevention because they would allow repeated measures from the same individuals for the examination of changes in health outcomes and costs over time. However, longitudinal studies often face challenges in measuring long‐term intervention effects due to attrition and changing environments.^54^, ^55^ Only one intervention (CORD) identified in this review adopted a longitudinal design to estimate the return on $1 invested. More exploration of longitudinal design is recommended to assist policymakers to make informed decisions about which interventions are the most cost‐effective or economically viable for preventing obesity in childhood.

Evidence of intervention effect on the primary outcome of body weight or BMI is also sometimes lacking. Results from our review demonstrate that the absence of intervention effectiveness in some cases has led to limited economic evidence being produced. For instance, the economic evidence is not available to date for multicountry OPIC project as planned^40^, ^41^ while several published studies from the project are available with mixed results (mostly not significant).^56^, ^57^, ^58^, ^59^ A recent paper from the WHO STOPs intervention also revealed that although the intervention was effective for the first 2 years, it became ineffective after 4 years.^60^ Such nonlinear responses to interventions impact on the ability to accurately model potential costs and benefits over a longer time period (beyond childhood), particularly in lieu of either good quality data on maintenance of intervention effect over time or rigorous sensitivity analyses varying effect maintenance assumptions in order to produce credible results. It also highlights the critical importance of intervention effect sustainability through the adoption of a life course approach to obesity prevention.

The findings from this review reveal that generating economic evidence of cost‐effectiveness for complex obesity prevention CBIs currently relies heavily on model‐based analyses. The time horizon of the modeled economic evaluation studies and protocols included in our review was similar, with five studies^18^, ^24^, ^34^, ^35^, ^39^, ^60^ estimating cost‐effectiveness over long periods of time (i.e., the lifetime), three of which were reported as cost‐effective.^18^, ^34^, ^35^, ^42^ These model‐based estimations provide crucial evidence to decision‐makers of the longer term benefits likely to result from a reduction of overweight and obesity in childhood. This longer time horizon is crucial, given that many of the benefits from prevention of chronic diseases in childhood may not become apparent until much later in life. Findings are, however, very sensitive to modeling assumptions, particularly around the maintenance of intervention effect,^18^, ^24^, ^34^, ^35^ as has been noted previously.^15^, ^61^ Several of the modeled economic evaluations included in our review provided sensitivity analyses, varying duration of intervention effect and assuming differing rates of intervention effect.^18^, ^24^ The study by Ananthapavan et al.^34^ incorporated a threshold analysis to assess the duration of effect required for the intervention to be cost‐effective. Based on the findings of our review, and the established importance of assumptions relating to duration of effect, we would recommend that this practice be adopted in all model‐based economic evaluation of CBIs for obesity prevention. In addition, there is a clear need for more comprehensive assessment and inclusion of the benefits of obesity prevention within the childhood to adolescent timeframe to be included into modeled economic evaluation of CBIs for obesity prevention. Notably, the most recent study modeled costs and benefits of the Romp & Chomp intervention to age 15 years,^42^ and there is clearly scope for the inclusion of similar short‐to‐mid‐term benefits to be included in more modeled economic evaluations.

To date, there is limited economic evidence that attempts to incorporate spillover effects, or the potential wider health benefits of CBIs for obesity prevention (e.g., productivity cost averted).^39^, ^62^ Failing to incorporate spillover effects (i.e., effects in others not specifically targeted by the intervention, such as families, parents, and other adults) may mean that the cost‐effectiveness of CBIs for obesity prevention is currently underestimated. For example, intervention activities of the OPIC project in Tonga often attracted the other age groups of the community; nonetheless, intervention effect beyond the target group was not captured.^63^ To date, little is known about possible ‘cross‐over’ effects or ‘contamination’ in large‐scale community‐based interventions, where participants in control communities may migrate toward intervention group participants,^53^ and this has also not been rigorously examined within the economic literature. Cost‐effectiveness may also be underestimated by not incorporating the value of benefits that may arise as a result of a community‐wide initiative to improve health and well‐being (e.g., productivity cost‐saving, the value of community engagement, capacity building, and social cohesion).

A recent review highlighted the methodological challenges of conducting economic evaluations incorporating the broader costs and benefits of prevention and health promotion interventions.^64^ The review recommended the ROI method to incorporate broader outcomes by analyzing views of multiple stakeholders and converting these in a monetary value (ratio).^64^ In recent years, the use of ROI has increased substantially while economically analyzing complex public health interventions by identifying benefits in terms of dollar value per 1$ investment (cost).^31^, ^64^ Recent evidence has highlighted the value of providing economic information in a way that is acceptable to communities, to build the investment case for prevention.^65^ The method was adopted by Coffield et al. in evaluating the SUS intervention and revealed cost‐saving outcomes.^31^ More research is therefore recommended using this methodology to economically evaluate obesity prevention CBIs.

Appropriate resource tracking is required for a comprehensive economic analysis, although this can be difficult for complex CBIs given the multisectoral, multiactor, dynamic nature of intervention.^10^ Sweeney et al. discussed the challenges of tracking resource utilization across complex interventions and suggested pragmatic solutions in their economic evaluation protocol for the WHO STOPs CBI^39^; however, it is not clear that adequate methodological solutions to the challenges of tracking resource utilization across complex interventions engaging multiple stakeholders have been found. While costing methods for complex interventions likely require a balance between the comprehensiveness of data, community sensitivity, and feasibility,^39^ this presents a serious challenge at the methodological level for conducting high‐quality, rigorous economic evaluations that adequately capture an intervention's costs and benefits. Communities must be resourced adequately to record and provide this information, or research support provided, and clear processes are required for identifying and recording resource utilization at multiple levels. Another important methodological consideration raised by Sweeney et al.^39^ was methods for attribution of costs, particularly when CBIs may involve actions that may be context dependent and across multiple sectors or carried out by multiple stakeholders. Clear decision rules are required to attribute these costs,^39^, ^66^ yet no guidance currently exists in terms of how these decision rules should be formulated and/or applied. Clearly, these are areas for future work.

### 4.1 Future research and policy implications of findings

**FIGURE 2:**
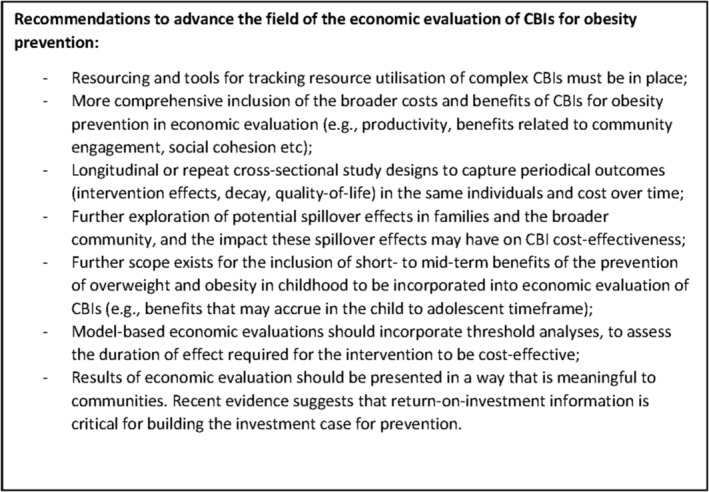
Recommendations for future research scope and policy implications.

The review uncovered several challenges in synthesizing economic evidence from the included interventions. These challenges include a lack of long‐term evidence on intervention effectiveness, reliance on model‐based studies, difficulties in tracking resources across multiple sectors, and a shortage of longitudinal studies to track costs and outcomes over time. As a result of these challenges, cost evidence was either reported to a lesser extent (e.g., OPAL) or not available in publications (e.g., OPIC). Based on these findings, a list of future research and policy implications has been included in Figure 2. The identified challenges underscore the need for more rigorous economic evaluations of interventions, particularly those involving multiple sectors. Additionally, greater attention should be paid to collecting long‐term outcome and cost data, as well as exploring innovative approaches to tracking resource utilization across sectors. These steps will help to improve the quality of economic evidence available to inform decision‐making in healthcare policy and practice.

### 4.2 Strengths and limitations

The major strengths of this review include the application of a systematic method following PRISMA guidelines and the most recent CHEERS checklist.^29^ Other significant strengths are the comprehensive search strategies, the use of a wide range of academic databases and the grey literature, and review by two independent reviewers. However, this review is limited to complex obesity prevention CBIs. The methods used to understand the cost‐effectiveness of complex CBIs outside child obesity prevention fall outside the scope of this review but may offer additional methodological insights. In addition, quality assessment of the included studies was not undertaken given the lack of a well‐validated and universally accepted tool for economic evaluation studies.^67^

## 5 CONCLUSIONS

The findings of this review indicate that there is ongoing interest in evaluating cost‐effectiveness of CBIs for childhood obesity prevention; however, available evidence is limited. The studies demonstrated mixed results and used widely varying methods. Complex CBIs are important for preventing childhood obesity, and reliable economic analysis, using standardized or comparable outcomes, is critical for the efficient allocation of scarce resources. This review further demonstrated that more research and methodological development are required to overcome challenges in accurately tracking resources and ensuring that the full breadth of potential benefits generated by CBIs for obesity prevention can be captured.

## AUTHOR CONTRIBUTIONS

Marufa Sultana and Vicki Brown conceptualized and designed the study with the inputs from all co‐authors. Marufa Sultana and Vicki Brown developed search strategies and undertook the searches and screening. Marufa Sultana extracted the data, and Marufa Sultana and Vicki Brown synthesized all data. Marufa Sultana drafted the manuscript under the supervision of Vicki Brown, and all authors critically reviewed the manuscript and provided expert opinion. All authors have read and approved the final version of the manuscript.

## CONFLICT OF INTEREST STATEMENT

No conflict of interest statement.

## Supporting information


**Table S1.** PRISMA Checklist 1.
**Table S2.** Systematic search strategies.
**Table S3.** Narrative summary of the included studies, according to CHEERS checklist (2022) item 4.

## Data Availability

The data are available within the manuscript and supplementary files.
