# Supplementary material for: A systematic review of economic evidence for community‐based obesity prevention interventions in children
Source: Obes Rev. 2023 Jun 12;24(9):e13592. doi: 10.1111/obr.13592 (PMC10909472; doi:10.1111/obr.13592)
Supplement: Supplementary file 1 — Table S1. PRISMA Checklist 1. Table S2. Systematic search strategies. Table S3. Narrative summary of the included studies, according to CHEERS checklist (2022) item 4. [file OBR-24-e13592-s001.pdf]

Supplementary Files

Title: A systematic review of economic evidence for community-based obesity prevention interventions in children

Marufa Sultana[1,2], Melanie Nichols[2], Marj Moodie[1,2], Steven Allender[2], Vicki Brown[1,2]

1 Deakin Health Economics, Institute for Health Transformation, School of Health and Social Development, Deakin University, Geelong, Victoria, Australia

2 Global Centre for Preventive Health and Nutrition (GLOBE), Institute for Health Transformation, School of Health and Social Development, Deakin University, Geelong, Victoria, Australia

Corresponding author: Marufa Sultana, Deakin Health Economics, Global Centre for Preventive Health and Nutrition (GLOBE), Institute for Health Transformation, School of Health and Social Development, Deakin University, Geelong, Victoria 3220, Australia. E-mail: m.sultana@deakin.edu.au

Table S1: PRISMA Checklist [1]

Section/topic	#	Checklist item	Reported on page #
**TITLE**			
Title	1	Identify the report as a systematic review, meta-analysis, or both.	1
**ABSTRACT**			
Structured summary	2	Provide a structured summary including, as applicable: background; objectives; data sources; study eligibility criteria, participants, and interventions; study appraisal and synthesis methods; results; limitations; conclusions and implications of key findings; systematic review registration number.	3
**INTRODUCTION**			
Rationale	3	Describe the rationale for the review in the context of what is already known.	4-5
Objectives	4	Provide an explicit statement of questions being addressed with reference to participants, interventions, comparisons, outcomes, and study design (PICOS).	6
**METHODS**			
Protocol and registration	5	Indicate if a review protocol exists, if and where it can be accessed (e.g., Web address), and, if available, provide registration information including registration number.	PROSPERO: CRD42021262817
Eligibility criteria	6	Specify study characteristics (e.g., PICOS, length of follow-up) and report characteristics (e.g., years considered, language, publication status) used as criteria for eligibility, giving rationale.	6-7
Information sources	7	Describe all information sources (e.g., databases with dates of coverage, contact with study authors to identify additional studies) in the search and date last searched.	6
Search	8	Present full electronic search strategy for at least one database, including any limits used, such that it could be repeated.	Page 6 in the main paper and supplementary file (table S2)
Study selection	9	State the process for selecting studies (i.e., screening, eligibility, included in systematic review, and, if applicable, included in the meta-analysis).	6-7
Data collection process	10	Describe method of data extraction from reports (e.g., piloted forms, independently, in duplicate) and any processes for obtaining and confirming data from investigators.	7-8

Data items	11	List and define all variables for which data were sought (e.g., PICOS, funding sources) and any assumptions and simplifications made.	7-8
Risk of bias in individual studies	12	Describe methods used for assessing risk of bias of individual studies (including specification of whether this was done at the study or outcome level), and how this information is to be used in any data synthesis.	Not conducted, reason added in the limitation section in page 20.
Summary measures	13	State the principal summary measures (e.g., risk ratio, difference in means).	NA
Synthesis of results	14	Describe the methods of handling data and combining results of studies, if done, including measures of consistency (e.g., I^2) for each meta-analysis.	NA
Risk of bias across studies	15	Specify any assessment of risk of bias that may affect the cumulative evidence (e.g., publication bias, selective reporting within studies).	NA
Additional analyses	16	Describe methods of additional analyses (e.g., sensitivity or subgroup analyses, meta-regression), if done, indicating which were pre-specified.	NA
**RESULTS**			
Study selection	17	Give numbers of studies screened, assessed for eligibility, and included in the review, with reasons for exclusions at each stage, ideally with a flow diagram.	8-9 & Figure 1
Study characteristics	18	For each study, present characteristics for which data were extracted (e.g., study size, PICOS, follow-up period) and provide the citations.	Table 2
Risk of bias within studies	19	Present data on risk of bias of each study and, if available, any outcome level assessment (see item 12).	NA
Results of individual studies	20	For all outcomes considered (benefits or harms), present, for each study: (a) simple summary data for each intervention group (b) effect estimates and confidence intervals, ideally with a forest plot.	10-15
Synthesis of results	21	Present results of each meta-analysis done, including confidence intervals and measures of consistency.	NA
Risk of bias across studies	22	Present results of any assessment of risk of bias across studies (see Item 15).	NA
Additional analysis	23	Give results of additional analyses, if done (e.g., sensitivity or subgroup analyses, meta-regression [see Item 16]).	NA

**DISCUSSION**			
Summary of evidence	24	Summarize the main findings including the strength of evidence for each main outcome; consider their relevance to key groups (e.g., healthcare providers, users, and policy makers).	16-18
Limitations	25	Discuss limitations at study and outcome level (e.g., risk of bias), and at review-level (e.g., incomplete retrieval of identified research, reporting bias).	19-20
Conclusions	26	Provide a general interpretation of the results in the context of other evidence, and implications for future research.	20
**FUNDING**			
Funding	27	Describe sources of funding for the systematic review and other support (e.g., supply of data); role of funders for the systematic review.	Title page (Acknowledgment section)

Table S2: Systematic search strategies

A systematic search of twelve databases was undertaken through November 2021, and updated on April 2022, to identify studies that fit the inclusion criteria. Search terms were adapted from the study by Flego et al.[2], and further developed in conjunction with a subject specific librarian. Search terms related to four key concepts: (1) study design: cost analyses or economic evaluations; (2) setting: community-based interventions; (3) outcome of interest: obesity; and (4) intervention: prevention interventions. Academic database search strategies with number of hits are given below: follow.

Academic databases:

Databases	Subject heading/Thesaurus/limiters	No. of hits
**Ebscohost:** Academic Search Complete;CINAHL Complete;EconLit;Global Health;MEDLINE Complete;APA PsycInfo;SPORTDiscus	(Cost OR "cost evaluat*" OR "cost analy*" OR "cost effectiv*" OR "cost benefit*" OR "cost utili*" OR "cost minimi*" OR "cost consequence" OR "economic* model*" OR "economic* evaluat*" OR "economic* analy*" OR "health economics") AND ("community based" OR "community wide" OR "community-based" OR "whole of community") AND (Obes* OR overweight OR "healthy weight" OR "healthy eating" OR "physical activity" OR "healthy lifestyle") AND (Prevent* OR Prevention)	892
**Embase**	('cost' OR 'cost'/exp OR cost OR 'cost evaluat*' OR 'cost analy*' OR 'cost effectiv*' OR 'cost benefit*' OR 'cost utili*' OR 'cost minimi*' OR 'cost consequence' OR 'economic* model*' OR 'economic* evaluat*' OR 'economic* analy*' OR 'health economics'/exp OR 'health economics') AND ('community based' OR 'community wide' OR 'community-based' OR 'whole of community') AND (obes* OR 'overweight' OR 'overweight'/exp OR overweight OR 'healthy weight' OR 'healthy eating'/exp OR 'healthy eating' OR 'physical activity'/exp OR 'physical activity' OR 'healthy lifestyle'/exp OR 'healthy lifestyle') AND (prevent* OR 'prevention' OR 'prevention'/exp OR prevention) AND [2006-2022]/py AND [english]/lim	254
**Scopus**	cost OR "cost evaluat*" OR "cost analy*" OR "cost effectiv*" OR "cost benefit*" OR "cost utili*" OR "cost minimi*" OR "cost consequence" OR "economic* model*" OR "economic* evaluat*" OR "economic* analy*" OR "health economics" AND "community	13,623

	based" OR "community wide" OR "community-based" OR "whole of community" AND obes* OR overweight OR "healthy weight" OR "healthy eating" OR "physical activity" OR "healthy lifestyle" AND prevent* OR prevention AND (LIMIT-TO (PUBYEAR , 2022) OR LIMIT-TO (PUBYEAR , 2021) OR LIMIT-TO (PUBYEAR , 2020) OR LIMIT-TO (PUBYEAR , 2019) OR LIMIT-TO (PUBYEAR , 2018) OR LIMIT-TO (PUBYEAR , 2017) OR LIMIT-TO (PUBYEAR , 2016) OR LIMIT-TO (PUBYEAR , 2015) OR LIMIT-TO (PUBYEAR , 2014) OR LIMIT-TO (PUBYEAR , 2013) OR LIMIT-TO (PUBYEAR , 2012) OR LIMIT-TO (PUBYEAR , 2011) OR LIMIT-TO (PUBYEAR , 2010) OR LIMIT-TO (PUBYEAR , 2009) OR LIMIT-TO (PUBYEAR , 2008) OR LIMIT-TO (PUBYEAR , 2007) OR LIMIT-TO (PUBYEAR , 2006)) AND (LIMIT-TO (LANGUAGE , "English"))	
**Pubmed**	((((Cost OR "cost evaluat*" OR "cost analy*" OR "cost effectiv*" OR "cost benefit*" OR "cost utili*" OR "cost minimi*" OR "cost consequence" OR "economic* model*" OR "economic* evaluat*" OR "economic* analy*" OR "health economics")) AND ("community based" OR "community wide" OR "community-based" OR "whole of community")) AND (Obes* OR overweight OR "healthy weight" OR "healthy eating" OR "physical activity" OR "healthy lifestyle")) AND (Prevent* OR Prevention) **Filters:** Abstract, Journal Article, English, from 2006 - 2022	289
**Health Technology Assessment Database**	(Cost OR "cost evaluat*" OR "cost analy*" OR "cost effectiv*" OR "cost benefit*" OR "cost utili*" OR "cost minimi*" OR "cost consequence" OR "economic* model*" OR "economic* evaluat*" OR "economic* analy*" OR "health economics") AND ("community based" OR "community wide" OR "community-based" OR "whole of community") AND (Obes* OR overweight OR "healthy weight" OR "healthy eating" OR "physical activity" OR "healthy lifestyle") AND (Prevent* OR Prevention) FROM 2006 TO 2022	01

**Cochrane library**	("cost" OR "cost evaluat*" OR "cost analy*" OR "cost effectiv*" OR "cost benefit*" OR "cost utili*" OR "cost minimi*" OR "cost consequence" OR "economic* model*" OR "economic* evaluat*" OR "economic* analy*" OR "health economics") AND ("community based" OR "community wide" OR "community-based" OR "whole of community") AND (Obes* OR overweight OR "healthy weight" OR "healthy eating" OR "physical activity" OR "healthy lifestyle") AND (Prevent* OR Prevention)	106

Grey literature: Systematic searches of the grey literature were also undertaken, using Google. The same search strategy was used through google to identify additional studies and no additional studies were identified over 10 pages. Next, a targeted search was undertaken by adding specific name of the CBI reported in Karacabyeli et al. [3] with relevant cost terms, for instance "Shape up in Somerville" AND Cost OR "cost evaluat*" OR "cost analy*" OR "cost effectiv*" OR "cost benefit*" OR "cost utili*" OR "cost minimi*" OR "cost consequence" OR "economic* model*" OR "economic* evaluat*" OR "economic* analy*" OR "health economics". Two reports and two academic papers were identified from this extensive grey literature search.

Table S3: Narrative summary of the included studies, according to CHEERS checklist (2022) item [4]

**1. APPLE (A Pilot Program for Lifestyle and Exercise)**	
**McAuley et al. 2009 [5]**	
Type of publication	Peer-reviewed economic evaluation
Publication title	Economic evaluation of a community-based obesity prevention program in children: the APPLE project
Study aim	To estimate the cost of APPLE intervention, its effects on weight and benefits in terms of health-related quality of life (HRQoL).
Country	New Zealand
Setting	Seven primary schools in Otago, New Zealand (intervention n=4; control n=3)
Design	Trial-based economic evaluation (CEA)
Perspective	Societal
Target population	Children aged 5-12 years
Sample size	n=279 (students of intervention schools); Analysis: Baseline N: I: C: 250:219<br>1 year N: I: C: 246:217; 2 year N: I: C: 151:136
Intervention	• Community Activity Coordinators' (ACs) support at intervention schools. ACs were supervised by a program coordinator. The main roles of the ACs included encouraging children to be more physically active, creating opportunities for physical activity at lunchtime and after school. Additional emphasis was on lifestyle-based activities rather than traditional sports. Extra sports and activity equipment was provided at interval and lunch time by the project.<br>• The intervention include nutrition-based component that included supplying a cooled water filter to intervention schools and free fruit for six months. A number of nutrition related resources were developed to reduce sugary drinks and to increase fruit and vegetable intakes (e.g., "APPLE Bites" which is a community-based resource developed for the project highlighting ideas, recipes, and tips for being more physically active and healthy eating while at home, card games and science lessons at school.
Comparator	No intervention
Time horizon	2-year intervention, 2-year follow up
Currency, price year and conversion	NZD, 2006, (NZ$1 = US$0.67 = UK£0.35 = EUR€0.52)
Discount rate	5% for costs
Measurement and valuation of resources and costs	Resource identification and measurement: Cost data collected prospectively. Only marginal costs of the project that were repeated in the future were included (i.e. project planning cost and cost for developing resources were not included). Costs were calculated using a conservative approach, but specific details not given on how costs and resources were identified (e.g. using trial records, data logs etc.).<br><br>Time costs related to the preliminary consultations (involved communities, schools) were included. Since the intervention was designed in a way that no additional workload was associated with teachers, therefore, time costs of the teachers were not included. The study also did not consider time costs of parents and children stating that measuring and valuing these costs were complex.

Cost categories	• Travel (vehicle petrol, lease, car parking)<br>• Time costs (Project coordinator, activity coordinators, training coordinators)<br>• Materials (dietary resource booklet, postage, stationery, phone, fax, stadiometer, scales, water filters, sports equipment, water bottles, vouchers and rewards, activity and food card game, photocopying, resource for teachers, advertising through radio ads etc).<br>• Overheads (average per diem cost of project coordinator, office space, rates and water, heating, photocopier).
Selection of outcomes	• BMI after 2 years of intervention, and 2 additional years of follow up.<br>• Kilogram of weight-gain prevented per child per year.<br>• Cost per kilogram of weight gain prevented.
Measurement of outcomes	Measured in duplicate at baseline, 1 and 2 years. BMI z-scores calculated according to 2002 CDC reference norms.[6]
Measurement and valuation of outcomes	The children's HRQoL was measured using the health utility index (HUI) (parental proxy), but this data was not used in a CUA.
Analytical method	Differences between HUI scores at baseline and 2 years analysed using chi-squared t-tests.<br>Within-trial CEA, cost per kg of weight gain prevented per child per year.
Rationale and description of model	Not applicable
Model assumptions	Not applicable
Characterising heterogeneity	Not explicitly mentioned
Characterising distributional effects	Not explicitly mentioned
Uncertainty analysis	Not explicitly mentioned
Approach to engagement with patients and others affected by the study	School children and staff, community via ACs
Sensitivity analysis	To determine the difference in weight (in kgs) between intervention and comparison groups if the APPLE intervention had been more or less successful at each point of time. The analysis used the confidence intervals of the differences in weight z-score.
Key findings (study parameters, incremental costs and outcomes)	**Effectiveness**<br>**At 2 year**<br>BMI z-score: Significantly lower in intervention children relative to control children by 0.26 units (95% CI: -0.32, -0.21).<br>Weight z-score by 0.18 units (95% CI: -0.22, -0.13).<br>**At follow-up**<br>BMI z-score: Significantly lower in intervention children relative to control children by 0.21 units (95% CI: -0.14, -0.22)<br>Weight z-score by 0.17 units (95% CI-0.11, -0.23).<br>• Differences in absolute weight in children after 4 years ranged between 1.0 and 2.0 kg in males and 1.1 and 1.9 kg in females aged 9-15 years.<br>• Non-significant difference in HUI scores.<br>**Cost**<br>After discounting, the total cost was estimated to be NZ332,952; NZ1,193 per child for 2 years.<br>**Cost-effectiveness**

	The cost per kilogram of weight-gain prevented over the 2 years of the intervention was NZ1,708 in 7-year old children (average weight gain prevented of 0.75kg) and NZ664 in 13-year old children (average weight gain prevented 1.93kg).
Sensitivity results	By the end of the intervention, the differences in weight ranged between 0.5 and 1.0 kg in case of youngest children and 1.4 and 2.4 kg in case of oldest children. In contrast, after 4 years of intervention, the differences in weight were mostly similar as observed at the end of the intervention (ranging between 0.6 kg and 2.8 kg), given that there was no direct intervention costs in follow-up period.
Heterogeneity	Not explicitly mentioned
Limitations	• Small sample size<br>• Lack of data on long-term effectiveness<br>• Only direct costs considered (perspective mentioned as societal but no family cost/other than project costs were reported).
Funding	Health Research Council, the National Heart Foundation, the Community Trust of Otago, the University of Otago, and the Otago Diabetes Research Trust
Conflict of interest	The authors declared no conflict of interest
**Health Technology Analysts, 2010 [7]**	
Type of publication	Report prepared for the Health Research Council of New Zealand
Publication title	Cost effectiveness report of public health interventions to prevent obesity. Report prepared for the Health Research Council of New Zealand (the APPLE project).
Study aim	The objective of the overall project was to estimate the cost-effectiveness of various public health interventions aimed at preventing obesity in New Zealand. APPLE was selected for analysis.
Country	New Zealand
Setting	Primary schools
Design	Modelled CUA
Perspective	Healthcare/funder
Target population	9 year old children, general NZ population
Sample size	NZ population
Intervention	The APPLE intervention for New Zealand population
Comparator	No intervention
Time horizon	Lifetime (up to 91 years)
Currency, price year and conversion	NZD, 2006, converted to 2010 NZD value
Discount rate	3.5% for both costs and outcomes
Measurement and valuation of resources and costs	Intervention costs estimated in McAuley et al (2009) were converted to annual cost per child and updated to 2010 NZD.<br><br>Annual health care costs of 'good health' and cost of illness data from the literature were used to estimate costs related to ongoing health care.
Cost categories	• Administrative costs<br>• Labour costs (part-time cost for project coordinator for two-years)<br>• Labour costs (full-time equivalent costs for activity co-ordinators for two years)<br>• Training costs<br>• Costs for project implementation (materials, equipment)<br>• Advertising costs

	• General overhead costs (e.g., office space, water, heating, insurance, phone etc.)
Selection of outcomes	QALYs
Measurement of outcomes	Impact of intervention on BMI from the literature (-0.2 reduction in BMI in Year 1, -0.4 in Year 2-5).
Measurement and valuation of outcomes	A comprehensive literature search on clinical and economic outcomes of interest was conducted to source each of the health states considered in the economic model to estimate the utility weights associated with the health states. These utility weights were applied to the health states in the model.
Analytical method	The economic model assigned costs and outcomes to each of the health states (e.g., utility weights in the base case, life years). The model calculated incremental cost-effectiveness ratios (ICERs) to determine cost-effectiveness of the intervention.
Rationale and description of model	The main rationale to conduct this analysis was to assist decision makers and to determine cost-effective investment in public health interventions for prevention of obesity and associated health problems for the population of New Zealand.<br>A simulation was performed to calculate the BMI of 10,000 individuals for each of the ethnic groups for each age (2 to 75 years). Each of the simulated individuals was categorised as 'being overweight', 'obese' or 'healthy' weight. According to age, BMI thresholds for each category was considered to accomplish categorisation. This simulation was also performed to the control arm (no intervention) of the economic model. The simulation was performed once only for each individual age.
Model assumptions	• The model estimated the proportion of 'overweight', 'obese' or 'healthy' weight individuals. To attain this, the effect of the intervention i.e., mean BMI was subtracted for each simulated individual for generating a new post-intervention cohort (10,000 individuals).<br>• The proportion of individuals from each weight category was then altered to indicate the anticipated distribution following the intervention.<br>• The model then estimated the probability of either staying in 'good health' or of contracting any of the selected fourteen obesity-related chronic illnesses (acute myocardial infarction; coronary heart disease; congestive heart failure; cerebrovascular accidence; arthrosis of the knee and hip; cancer of the rectum, colon, breast, prostate, kidney, endometrium) respective to age, in each yearly cycle (incidence rates were taken from general NZ population relative risks were sourced from the literature).<br>• The utility weights used in the model were derived using a multi-attribute utility instrument (MAUI). This is a well-accepted instrument that undergone a process of validation over time. A priori preference was also considered for further internal consistency along with the source of the utility weights used in the model, if available.
Characterising heterogeneity	Not explicitly mentioned
Characterising distributional effects	Not undertaken
Uncertainty analysis	Not undertaken
Approach to engagement with patients and others	As per AAPLE intervention (school children, staff and community).

affected by the study	
Sensitivity analysis	One-way: Variation in age, cost of intervention, discount rate, rate of decay, the degree of up taking the interventions and the effectiveness estimates among lower SE group, shortening the time horizon to age 60
Key findings (study parameters, incremental costs and outcomes)	**Effectiveness**<br>QALYs: Intervention Vs Control: 26.185 vs 26.178<br>Intervention effect: -0.2 reduction in BMI in Year 1, -0.4 in Year 2-5.<br>**Cost**<br>Annual cost per participant NZD704.73<br>Intervention: Lifetime health care costs of $54,653<br>Control: Lifetime health care costs of $53,260<br>**Cost-effectiveness**<br>Incremental cost: NZD1,393, incremental benefit (QALY): 0.007<br>ICER: NZD 205,101 per QALY gained
Sensitivity results	• One-way, tested different variables (e.g. age, % change of costs, intervention decay 1%-10%<br>• Results most sensitive to rate of decay
Heterogeneity	Not discussed
Limitations	• Lack of long-term follow-up data on the effectiveness of the interventions on BMI.<br>• The model assumed long-term effect of the interventions to all participants regardless of their baseline BMI.<br>• Lack of intervention cost information are subject to varying degrees of uncertainty.<br>• The model assumption considered 'good health' for all individuals and health state as 'perfect health', in addition, no disutility was applied with age.<br>• Use of Dutch data to inform the model (e.g. population incidence rates).
Funding	Report prepared for the Health Research Council of New Zealand
Conflict of interest	NS
2. **Be Active Eat Well (BAEW)**	
Moodie et al. 2013 [8]	
Type of publication	Peer-reviewed economic evaluation
Publication title	The cost-effectiveness of a successful community-based obesity prevention program: the Be Active Eat Well (BAEW) program
Study aim	To examine the cost-effectiveness of BAEW intervention for Australian children aged 4-12 years during the period of project funding.
Country	Australia
Setting	Primary schools in a rural town (Colac) in Victoria, Australia.
Design	Within trial and modelled CUA; retrospective
Perspective	Societal
Target population	Children 4-12 years
Sample size	Within trial: Intervention: 1,001; Comparator: 1,183.<br>Modelled: For one year considering that the intervention would be implemented by 10% of Australian primary schools (n=181,212 children across 656 schools).
Intervention	• The intervention was multifaceted, complex and targeted whole-of-community. The primary setting of the intervention was primary schools (six primary schools and four preschools in Colac city).<br>• The main intervention component included evidence-based behaviour change: reduced consumption of sugar sweetened drinks, increased

	consumption of water, increase intake of fruit and vegetables, reduction of television viewing, reduced consumption of energy dense snacks; increased active play after school and at weekends; and increased active transport to schools.<br>• To facilitate the BAEW intervention, Colac area was awarded AUD100,000 per year contract for four years. The local health services of Colac implemented the intervention.
Comparator	Current practice, defined as any initiatives (school or non-school) introduced into the school environment to encourage healthy eating, physical activity or to reduce childhood obesity, over and above normal curriculum activities.
Time horizon	3-year for intervention (mid 2003 to mid 2006), modelled for lifetime
Currency, price year and conversion	AUD, 2001, converted to 2006 value
Discount rate	3% for both costs and benefits
Measurement and valuation of resources and costs	• Pathway analysis was used to derive resource used related to the intervention[11]<br>• Detailed data on process evaluation was collected prospectively during the program. The comprehensive process evaluation data and intervention related reports enabled the relevant cost data extraction with details (e.g., cost of each of the program activities, invested personnel time for the project). The additional cost information was further achieved through financial documents, interviews with key stakeholders related to the program, and interviews with relevant personnel from each participating primary school.<br>• Cost information for current practice was obtained from control schools through interviewing the principals and respective staff (who were present during the time of the BAEW program), school newsletters and reports.<br>• Costs were measured in Australian dollars (AUD1.00 = USD0.748447 at 1 July 2006) and costs were adjusted using the relevant consumer price index to actual prices for the 2006 reference year. Unit prices were obtained from the most up-to-date and recognized sources.
Cost categories	• Materials (catering, paper/printing costs for training resources)<br>• Travel ($/km, bus hire)<br>• Time – (additional time cost of staff, parents, and volunteers associated with the intervention i.e., delivering or attending intervention activities.<br>• Unit costs per hour for volunteer/parent, School Principal, Assistant School Principal, Teachers, School Nurse, Canteen Attendant, Social Marketing Consultant, Casual Research Assistant, Chief Executive Officer, Public Servants, Community Development Officer, Food Service Chef, Manager Nutrition and Dietetics, Dietitian, Paediatrician, General Practitioner, Practice Nurse, Retailers, Administrative and Clerical Employees.<br>• Cost-savings from diseases averted.<br>• Key costs that were excluded: Time cost of students to participate in intervention activities, costs associated with any extra activities; and the costs of changing eating patterns or physical activity of participating families.

Selection of outcomes	Change in BMI, AUD per unit of body mass index (BMI) saved, and Disability-adjusted Life Years (DALYs) saved over the lifetime of the cohort.
Measurement of outcomes	Taken from effectiveness study, [11] the study used a quasi-experimental, and longitudinal design to measure anthropometric and demographic data of participants at baseline and the end of the intervention.
Measurement and valuation of outcomes	BMI reduction among intervention group was converted to saving in terms of DALYs using the model developed ACE-Obesity study. [10] The BMI used in the ACE-Obesity model to avert DALY commences at age 5, therefore, the DALYs averted for children <5 years were calculated in this model by running the relevant BMI savings through the age 5-9 years component of the model.
Analytical method	DALYs averted were calculated as the difference in future mortality and morbidity outcomes between the intervention scenario and current practice.
Rationale and description of model	The rationale of conducting CUA was to facilitate comparison of BAEW intervention with the interventions evaluated in the ACE-Obesity study. Consequently, the intervention was modelled for one year at a national level assuming that 10% of Australian primary school will take-up the intervention. The reduction in BMI from the intervention was converted to a saving in terms of DALYs using a model developed for the ACE-Obesity study. [10]
Model assumptions	• The impact of the intervention in relation to the BMI distribution was determined using potential impact fractions (PIFs), defined as the proportional change in expected disease or death, that is attributable to a change in exposure to the risk factor in the population. The PIFs included diseases were ischaemic heart disease, hypertensive heart disease, ischaemic stroke, type 2 diabetes, osteoarthritis, colon cancer, endometrial cancer, postmenopausal breast cancer, and kidney cancer. • The model considered the current prevalent cohort of children and adolescents aged between 5 and 19 years and follows them for their remaining life span until death (or age 100 years).<br>• The model is based on 2001 population, epidemiology, and cost of the diseases, therefore, there is a mismatch between the reference year used for costing (2006) and for DALY and cost-offset calculations (2001).
Characterising heterogeneity	Not explicitly mentioned
Characterising distributional effects	By gender and age categories
Uncertainty analysis	Probabilistic uncertainty analysis to present 95% UI around the health benefits (triangular distribution), cost offsets (triangular distribution) and ICERs, using @RISK software (https://www.palisade.com/risk/) and Monte Carlo simulations (6,000 iterations). Resource use and related costs were based on detailed process evaluation data, consequently, uncertainty distributions were not attached to intervention costs.
Approach to engagement with patients and others affected by the study	School children, families, school staff, communities
Sensitivity analysis	Different scenarios were tested to examine what effect component would need to be lost to make the intervention not cost-effective. Another

	scenario also tested to examine the intervention impact if only 50% of children received the benefit.
Key findings (study parameters, incremental costs and outcomes)	**Effectiveness**<br>• Lower increases in BMI for the children who received BAEW intervention compared to comparison group [BMI scores -0.28 (95% CI: -0.7; 0.15)]. The difference was not statistically significant.<br>• Intervene children gained less weight −0.92 (−1.74; −0.11), lower increases in waist circumference −3.14 (−5.07; −1.22), BMI z-score −0.11 (−0.21 to−0.01) and waist/height ratio −0.02 (−0.03; −0.004) compared to the comparison group.<br>• For Colac, 547(−104; 1209) BMI units were saved as a result of the intervention which translated to 10.2 (−0.19; 21.58) DALYs averted.<br>• When modelled, the intervention resulted in savings of 82,899 BMI units and 1521 DALYs.<br>**Cost**<br>• Average annual intervention cost: AUD61,200 per school community.<br>• Personnel time covered 64.1% of the total costs.<br>• For strategic objectives, the major part (42.2%) was spent on the active play.<br>• 60% of cost borne by health sector; 25% by local government; 12% by recreation and sport; 1.5% by education sector; <1% by commercial and other sectors respectively.<br>• The BAEW program was cost-saving given that every AUD1.00 of funding invested generated AUD2.80 worth of activity at the community level (cost of evaluation was not included); furthermore, AUD3.35 generated for every AUD1.00 invested by the community (cost of evaluation was included).<br>• For Colac, modest cost-offsets were estimated (AUD27,311 (95% UI -AUD1,803; AUD58,242).<br>• When modelled (for 10% of Australian primary schools), the result showed modest cost-offsets of AUD4.1M.<br>**Cost-effectiveness**<br>• For Colac, the BAEW intervention was cost-effective with an estimated net cost per DALY saved of AUD29,798 (dominated; AUD0.26M).<br>• There was a 73.2% chance that the intervention would be cost-effective meaning that cost less than the Australian benchmark for cost-effectiveness of AUD50 000 per DALY saved.<br>• When modelled (10% of Australian primary schools), the BAEW obesity prevention intervention was cost-effective with net cost per DALY saved of $20,227 (dominated; $0.20M).
Sensitivity results	If the intervention benefit was received by 50% of the children (participating schools), the intervention remains cost-effective with a considerably higher ICER (just below the cost-effectiveness threshold). On the other hand, if the populations receiving intervention benefits remained unchanged but the maintenance of intervention effects was 50% over time, the intervention was not cost-effective. Study mentioned that at least 70% of the intervention effect would need to be maintained to make sure that the intervention remained cost-effective.
Heterogeneity	Not explicitly mentioned

Limitations	• Assumption of 100% maintenance of intervention effect for base-case analysis.<br>• The benefit calculation in terms of DALYs averted was conservative given that the DALYs arising from obesity-related diseases would be greater if modelled for the 2006 reference year instead of 2001.
Funding	National Health and Medical Research Council project grant (479510)
Conflict of interest	The authors declared no conflict of interest
**Health Technology Analysts (report), 2010 [7]**	
Type of publication	Report prepared for the Health Research Council of New Zealand
Publication title	Cost effectiveness report of public health interventions to prevent obesity. Report prepared for the Health Research Council of New Zealand (The BAEW project)
Study aim	The objective of the overall report was to estimate the cost-effectiveness of different public health intervention related to obesity prevention in New Zealand. BAEW was selected for analysis.
Country	New Zealand
Setting	Primary schools
Design	Modelled CUA
Perspective	Healthcare/funder
Target population	8 year old children, general NZ population; also modelled for Maori population and Pacific population
Sample size	Modelled on NZ population
Intervention	BAEW intervention if applied for NZ population
Comparator	Current practice as per curriculum
Time horizon	Lifetime (up to 92 years)
Currency, price year and conversion	NZD, 2006, converted to 2010 value
Discount rate	3.5% for both costs and outcomes
Measurement and valuation of resources and costs	States that intervention costs were based on the report by Sanigorski et al (2008) [11]<br>Cost estimates were adjusted by assuming:<br>• The project grant money and funding would be used for implementing the BAEW intervention.<br>• 25% of the total funding would be used to set up the program, research and development.<br>• Overheads costs reported in the project funding documents were included.
Cost categories	Not separately described
Selection of outcomes	QALYs
Measurement of outcomes	Impact of intervention on BMI from the literature (-0.3 reduction in BMI year 3).
Measurement and valuation of outcomes	A comprehensive literature search on clinical and economic outcomes of interest was conducted to source each of the health states considered in the economic model to estimate the utility weights associated with the health states. These utility weights were applied to the health states in the model.
Analytical method	The economic model attributed costs and outcomes (life years and, in the base case, utility weights) to each of the health states. Calculated incremental cost-effectiveness ratios (ICERs) as a measure of cost-effectiveness.

Rationale and description of model	The main rationale to conduct this analysis was to assist decision makers and to determine cost-effective investment in public health interventions for prevention of obesity and associated health problems for the population of New Zealand for BAEW intervention.<br>A simulation was performed to calculate the BMI of 10,000 individuals for each of the ethnic groups for each age (2 to 75 years). Each of the simulated individuals was categorised as 'being overweight', 'obese' or 'healthy' weight. According to age, BMI thresholds for each category was considered to accomplish categorisation. This simulation was also performed to the control arm (no intervention) of the economic model. The simulation was performed once only for each individual age.
Model assumptions	• Each simulated individual was categorised as being overweight, obese or 'healthy' weight.<br>• Estimated BMI had a lognormal distribution.<br>• The same simulation method was performed for control population assuming 'no intervention' was provided as the BMI data provided simulation method was applied for control, assuming no intervention as the BMI data provided denotes the current status quo.<br>• As the model extended over time, individuals were assumed to change BMI status as per the simulation model.<br>• In order to estimate the proportion of 'overweight', 'obese' or 'healthy' weight individuals, the effect of the intervention i.e., mean BMI was subtracted for each simulated individual for generating a new post-intervention cohort (10,000 individuals).<br>• Dutch population survey data was used in order to estimate the likelihood of either staying in 'good health' or having any of the fourteen obesity-related chronic illnesses.<br>• Mortality was estimated in Each yearly cycle was considered to estimate mortality using New Zealand life tables
Characterising heterogeneity	Not explicitly mentioned
Characterising distributional effects	NZ general population, Māori population, Pacific population
Uncertainty analysis	Not undertaken
Approach to engagement with patients and others affected by the study	As per BAEW project (schools, families, communities).
Sensitivity analysis	One-way: Variation in age, cost of intervention, discount rate, rate of decay, the degree of up taking the interventions and the effectiveness estimates among lower SE group, shortening the time horizon to age 60
Key findings (study parameters, incremental costs and outcomes)	**Effectiveness**<br>• -0.3 reduction in BMI year 1-5<br>• QALYs: Intervention Vs Control: 25.645 vs 25.639<br>**Cost**<br>• Cost per participant NZD285.48<br>• Intervention: Lifetime health care costs of $48,404<br>• Control: Lifetime health care costs of $47,569<br>**Cost-effectiveness**<br>General population<br>• Incremental cost: NZD834, increase QALY: 0.005

	• ICER: NZD168,391 per QALY gained<br>MAORI population<br>• ICER: NZD123,536 per QALY gained<br>PACIFIC population<br>• ICER: NZD154,178 per QALY gained
Sensitivity results	Highly sensitive to the rate of decay of the intervention effect size. For example, an increase in the rate of decay from 1% to 10% per annum increased ICER by 1,171% for general NZ population.
Heterogeneity	Not explicitly mentioned
Limitations	• Lack of long-term follow-up data on the effectiveness of the interventions on BMI.<br>• The model assumed long-term effect of the interventions to all participants regardless of their baseline BMI.<br>• Lack of intervention cost information are subject to varying degrees of uncertainty.<br>• The model assumption considered 'good health' for all individuals and health state as 'perfect health', in addition, no disutility was applied with age.<br>• Use of Dutch data to inform the model (e.g. population incidence rates).
Funding	Report prepared for the Health Research Council of New Zealand
Conflict of interest	NS
**3. Childhood Obesity Research Demonstration (CORD)**	
**O'Connor et al. (2014)** [12]	
Type of publication	Peer-reviewed protocol, including costing and cost-effectiveness protocol
Publication title	Childhood Obesity Research Demonstration Project: Cross-Site Evaluation Methods
Study aim	To describe the plan and general framework of comprehensive evaluation of the CORD project through The CORD Evaluation Center (EC-CORD)
Country	USA
Setting	Primary healthcare clinics, education centres, and schools of different states of USA (Massachusetts, California and Texas).
Design	Protocol for cost and cost-effectiveness analyses alongside the trial
Perspective	Not clearly stated
Target population	Children aged 2-12 years
Sample size	Target: over 2000 families
Intervention	• Primary prevention encompasses early care and education and school-based programs, which accentuate and support healthy eating and physical activity (PA), together with the other community level programs for obesity prevention. Establishing opportunities and policies for healthy eating and PA create supportive environments to the family and community, with messages that strengthen and complement the behavioural targets of the interventions. [13]<br>• The intervention also included secondary prevention program that targeted overweight or obese children and their families. The secondary prevention was more intensive and include objectives to improve dietary intake and increase PA through healthcare and public health efforts. These secondary interventions were implemented in small group of people and/or one-on-one, with a particular focus on food and PA related skills and self-efficacy. Families are observed and connected to the healthcare system/facility through community health workers. [13]

Comparator	Comparison community with similar sociodemographic characteristics with no CORD supported intervention. [12, 13]
Time horizon	Two-year intervention
Currency, price year and conversion	Not explicitly mentioned
Discount rate	Not explicitly mentioned
Measurement and valuation of resources and costs	• Detailed cost information related to the intervention development and implementation such as training of the providers, cost of delivering the intervention in selected settings, cost of equipment and supply as well as other costs would be collected in a consistent manner.<br><br>• The project team will compile and code all cost data for each activity using standard method of cost-categorisation (e.g., labor, in-kind). EC-CORD will collect information from financial reports, budget sheets, budget sheets, and records from each of the CORD project to develop a standard format for cost data. For example, the projects from CORD intervention will track cost data per cost category, expenditure for specific intervention activity (e.g., staff training, education to families, equipment) and cost data for specific settings (e.g., total costs for personnel). These cost information will be used to assess costs of intervention, replication of the project, scalability and sustainability of the program. All costs will be assessed at different points of time (longitudinally).
Cost categories	The project team will compile and code all cost data for each activity using standard method of cost-categorisation (e.g., labor, in-kind, purchases).
Selection of outcomes	Weight-related changes: height, weight, BMI<br>**Behaviours**<br>• Fruit and vegetable consumption<br>• Water consumption<br>• Consumption of sugary beverage<br>• Physical activity<br>• Sleep time<br>• Screen time<br>• Quality of life<br>• Net benefit
Measurement of outcomes	Comprehensive longitudinal models will be used that will take into account the heterogeneity to assess the benefits of the CORD project. Outcome data for intervention participants (children and parents) are gathered through longitudinal data collection and a mix of longitudinal and cross sectional data are collated for comparison groups. Clinical data are collected at 0 an 12 months of analysis.
Measurement and valuation of outcomes	The PSE tools (a set of 35 A set of 35 collective measures will be used for the impact evaluation and to allow a uniform way to determine socio-demographic characteristics and common outcomes measures of the participants of the CORD project. The included questions were derived from validated instruments [14]. Preference based outcome measurement was not stated.
Analytical method	The benefits of the CORD project will be attained as per dollar invested in each setting and across benefits that can be gained per dollar invested in each setting and across the communities. Finding will inform the extent of

	which interventions would be regarded as being cost-effective in terms of costs and associated changes for the interventions (e.g., child activity, dietary behaviours, weight and quality of life measures).
Rationale and description of model	Not applicable
Model assumptions	Not applicable
Characterising heterogeneity	Will be considered
Characterising distributional effects	Age sub-groups: 2–5, 6–8 and 9–12 years
Uncertainty analysis	Not explicitly mentioned
Approach to engagement with patients and others affected by the study	Schools, communities, stakeholder groups
Sensitivity analysis	Not explicitly mentioned
Key findings (study parameters, incremental costs and outcomes)	Not applicable
Sensitivity results	Not applicable
Heterogeneity	Use of longitudinal model will account the heterogeneity in populations, geographical locations, intervention settings, socio-political environments, measurement methods, and timelines.
Limitations	Not discussed
Funding	Centers for Disease Control and Prevention (CDC), Atlanta, GA (grant R06/CCR121519-01)
Conflict of interest	No competing financial interests exist
**4. Community-based intervention (ACE Obesity Policy study)**	
Ananthapavan et al. 2019 [15, 16]	
Type of publication	Peer-reviewed economic evaluation
Publication title	Cost-effectiveness of community-based childhood obesity prevention interventions in Australia
Study aim	The review of the literature followed by meta-analysis to synthesize evidence and to assess cost-effectiveness of community-based interventions for obesity prevention in Australian settings.
Country	Australia
Setting	Australian setting
Design	Modelled CUA
Perspective	Limited societal
Target population	5-18 years
Sample size	2010 Australian population (primary and secondary school children)
Intervention	A hypothetical CBI was designed from the literature review that is appropriate for Australian setting. The hypothetical CBI was then costed. A generic programme was defined as “community-led obesity prevention actions administered within Local Government Areas (LGAs) across Australia”.
Comparator	Current practice
Time horizon	Intervention for three years. For the estimation of healthcare cost offset and long-term heal benefits of the intervention, lifetime time horizon (100 years) was considered.

Currency, price year	AUD, 2001, converted to 2010 value
Discount rate	3% for costs and benefits
Measurement and valuation of resources and costs	• The costs borne by different stakeholders within the community were estimated assuming the intervention was in 'steady state' (excluding research and development costs and assuming full effectiveness potential).<br>• The resource use and the unit costs for implementing the hypothetical CBI were sourced from trial cost data and from literature review. Common CBI related activities were determined by literature review and then costed to estimate average cost per activity (e.g., average cost per activity related to nutrition and PA strategy, the average cost of any physical/environmental change to school (e.g., garden, water fountain), and average cost of any change within the community (e.g., food-quality in local restaurants).<br>• The cost of promotion activities and central co-ordination were also estimated. Cost of healthcare was obtained from literature.
Cost categories	Time costs of personnel, with volunteer time cost valued at 30% of average wage.<br>Healthcare cost-savings from diseases averted.
Selection of outcomes	HALYs
Measurement of outcomes	The effectiveness of the intervention in BMI z-scores was informed by a meta-analysis and translated to a change in BMI for individual age and sex groups from 5–18 years.
Measurement and valuation of outcomes	Total HALYs included the immediate health-related quality-of-life improvements in children stemming from changes in BMI status from the literature.
Analytical method	Monte-Carlo simulations were used to calculate the mean incremental costs of the intervention compared with the comparator, which was divided by the mean incremental health benefits. Incremental cost-effectiveness ratios (ICERs) were calculated using the ratio of means approach. The intervention was judged to be cost-effective if the ICER was less than the commonly used willingness to pay threshold for Australia of AUD50,000 per HALY gained.
Rationale and description of model	A multiple cohort Markov model was used to estimate the short and long-term health outcomes resulting from changes in BMI. The model was used in several economic evaluations of obesity prevention interventions as part of a priority-setting study. [17-19]
Model assumptions	The model estimated the incidence, prevalence, and mortality of nine diseases causally related to elevated BMI over the life course (type 2 diabetes, stroke, hypertensive heart disease, ischaemic heart disease, osteoarthritis of the hip and knee, and several cancers (kidney, endometrial, breast, and colorectal). Each of these diseases were modelled with four health states (healthy, diseased, dead due to disease, dead from other causes). Transitions between states were determined by incidence (calculated using potential impact fractions) using relative risk of disease related to BMI, prevalence case fatality rates and all-cause mortality from the literature. Health states were weighted using disability weights from the literature. The time spent in each health state was aggregated to estimate HALYs. Total HALYs also included the

	immediate health-related quality-of-life improvements in children stemming from changes in BMI status from the literature.
Characterising heterogeneity	Reported
Characterising distributional effects	By age groups: Under 5 years, 5‐11 years old, 12‐18 years old, 5‐18 years old
Uncertainty analysis	Second-order parameter uncertainty analyses were undertaken applying Monte-Carlo simulation using the Excel. add-in software, Ersatz version 1.35 (EpiGear International 2016).
Approach to engagement with patients and others affected by the study	Communities, stakeholder groups
Sensitivity analysis	Extensive one-way sensitivity analyses and scenario analyses. The key variables assessed included the effect size, target population (limiting to primary school children only), and intervention intensity. Threshold analysis was undertaken to assess the duration of effect required for the intervention to be considered cost-effective. The intervention's cost-effectiveness inclusive of evaluation<br>costs was also tested.
Key findings (study parameters, incremental costs and outcomes)	**Effectiveness**<br>• Small but significant difference in BMI z-score favouring the CBI community compared with the control community for the whole targeted population aged 5–18 years (MD − 0.07 (95% UI: − 0.13 to − 0.01)); heterogeneity was high (I2 = 86%).<br>Subgroup analysis of intervention effectiveness:<br>• Children aged 5–11 years resulted in a larger effect size (MD − 0.12 (95% UI: − 0.23 to − 0.01))<br>• (12–18 year age group) showed that CBIs were not effective<br><br>**Costs**<br>Of the six intervention components costed, half of the total cost was attributed to physical activity strategies (AUD377,084 (95% UI: AUD339,542 to AUD412,457)) and nutrition strategies (AUD403,290 (95% UI: AUD341,537 to AUD464,420)) implemented within schools.<br>• The average cost per child over the 3-year intervention was ~AUD499. Each CBI was estimated to cost ~AUD1.52 million (M) (per LGA).<br>• The largest proportion of costs were borne by local government, including local health services (59%). Individuals and families contributed up to 18% to the total cost. Although the majority of strategies were implemented within schools (82%), only 20% of the total costs accrued to the education sector.<br><br>**Cost-effectiveness**<br>Across Australia, the intervention would reach over 2.3 million students across 5,913 government schools, resulting in 51,792 (95% UI: 6816 to 96,972) HALYs gained. Healthcare cost offsets were estimated to be AUD452M, resulting in a net cost of AUD426M (95% UI: $3M to $823M) and an ICER of AUD8155 per HALY gained (95% UI: AUD237 to AUD81,021). The probability of the intervention being cost-effective was 95%. The majority of ICER iterations (98%) fall in the northeast quadrant of the cost-effectiveness plane.

	• The financial contribution of families was relatively high ($92 per child)
Sensitivity results	Scenario analyses: The intervention remained cost-effective when the intervention intensity and the effect size were varied. If only primary schools implemented the CBI, the intervention was dominant (95% UI: dominant to AUD30,448) as a result of the larger effect size in younger children. When the intervention effect was decayed by 5% each year, the CBIs were no longer cost-effective. The threshold analysis showed that the intervention effect would need to last for 29 years for the intervention to be considered cost-effective.
Heterogeneity	High for outcomes
Limitations	• High heterogeneity<br>• The use of a limited societal perspective meant the downstream costs (and benefits) to the many different stakeholders were not fully captured.<br>• It was assumed that the changes in BMI as a result of the intervention would be maintained over the lifetime of the targeted population.
Funding	The work was funded by a National Health and Medical Research Council (NHMRC) Centre of Research Excellence (CRE) on Obesity Policy and Food Systems (Grant number 1041020).
Conflict of interest	None declared
**5. Fun 'n healthy in Moreland!**	
Waters et al. (2019)[20]	
Type of publication	Peer-reviewed paper, including cost analysis
Publication title	Cluster randomised trial of a school-community health promotion and obesity prevention intervention: findings from the evaluation of *fun 'n healthy in Moreland*!
Study aim	To improve child adiposity, school policies and environments, parent engagement, health behaviours and child wellbeing. The study evaluated the physical, environmental, social, behavioural and financial impacts and outcomes of the interventions
Country	Australia
Setting	The City of Moreland
Design	Cost analysis alongside trial
Perspective	Not clearly stated
Target population	Primary school aged children, prep to grade 6 (4 years to 13 years)
Sample size	At baseline 1628 students from 11 comparison school; 1,594 students from 12 intervention schools.<br>At follow-up, 1,539 (50.1%) eligible students from 10 comparison schools and 1,426 (48.5%) eligible students from 12 intervention schools
Intervention	Schools were supported to develop fun 'n healthy programs according to the fixed requirement of a whole school combined focus on increasing fruit, vegetable and water consumption, increasing physical activity and encouraging positive self-esteem in children. Within the intervention schools, the school community determined the exact content of the program strategies, based on interventions that had demonstrated evidence of implementation or success in previous studies, or innovative programs which had a strong likelihood of success. The fun 'n healthy in Moreland! study offered schools the support of Community Development Workers (CDWs) for the 3.5 year intervention period.
Comparator	Comparison group is exposed to background activities in the region with no specific intervention strategies instituted
Time horizon	Not stated

Currency, price year	AUD, 2004-2005, converted to 2009 value
Discount rate	5% for costs
Methods for estimating resource use and costs	A costing of the resources invested in the intervention. Costs incurred across all schools were split equally between the intervention schools. School-level costs were split equally across the student population.
Cost categories	• Time costs (CDW)<br>• School resources<br>• Parent expenses
Choice of health outcomes	• BMI z-score (primary)<br>• Fruit and vegetable intake and sweet drink consumption<br>• Participation in sedentary activity (SA), physical activity (PA) and activity intensity<br>• Child experience<br>• Impacts on the school, home and community environments
Measurement of effectiveness	• BMI z-score calculated using direct measure of child height and weight to generate BMI, and then z-scores against the WHO reference curves [21].<br>• Fruit and vegetable intake and sweet drink consumption by parent report through parent questionnaires covering issues such as family food habits [22] and<br>usual intake of fruit, vegetable, dairy and drink consumption [23]; by child report through child questionnaire assessing food behaviours [24]; by Direct Assessment of school foods and by 24-h food record [25].<br>• Participation in SA, PA and activity intensity was measured by Parental and child report in parent and child questionnaires covering issues such as family physical activities<br>• Child experience by child-report through child questionnaire of quality of life using the 10-item version of the international self-reported measure of quality of life, KidScreen [26]; and by child focus groups to explore children's concepts of health and strategies to promote health in the home and school environments<br>5. School reported audit, Principal exit interviews, Teacher-reported school- and class-based nutrition and physical activity initiatives, Observational measure: SOPLAY (System for Observing Play and Leisure Activity in Youth).
Measurement/valuation of preference based outcomes	Not applicable
Analytical method	Intervention effects are estimated based on the intention-to-treat (ITT) principle. Descriptive adiposity scores were generated using WHO cut points. continuous outcomes (e.g. bmi z-score), the intervention effect was estimated using random effects linear regression models fitted by maximum likelihood estimation<br>to allow for clustering. For dichotomous outcomes, marginal logistic regression models were fitted using generalized estimating equations with information sandwich ("robust") estimates of standard error. Models were also adjusted for child age and sex, socio-economic position (measured by maternal<br>education, residential SEIFA (Australian Bureau of Statistics Socio-economic Index for Areas (SEIFA) index of relative socioeconomic disadvantage), and ethnicity (only English spoken at home). Statistical

	analyses were conducted with STATA 10.1 (Stata Corp LP, College Station, Tex).<br>Cost was calculated for whole intervention period and then cost per school as well as cost per patient was calculated.
Choice of model	Not applicable
Model assumptions	Not applicable
Characterising heterogeneity	Not explicitly mentioned
Characterising distributional effects	Not explicitly mentioned
Uncertainty analysis	Not applicable
Sensitivity analysis	Not undertaken
Key findings (study parameters, incremental costs and outcomes)	**Effectiveness**<br>• No significant difference was observed for mean BMI z score between the two groups (mean difference: -0.05, 95% CI: 0.019 to 0.08, p = 0.44).<br>• Children attending intervention schools were also more likely to include plain water in their lunch box (OR 1.82 [95% CI 1.05–2.78], p = 0.03)<br>• No intervention effect on PA<br>• Environmental: Schools chose to expand their canteen policy to include a broader school-wide healthy eating policy to include strategies such as healthy fundraising, drink water policies and replacement of confectionary as in-class rewards.<br>**Cost**<br>The total estimated cost (discounted) of a community development worker providing external support to schools was $55,868 per school over the full period of the study or $229 per student. There was no associated increase in parent-reported costs to families/$65 per child per year.
Approach to engagement with patients and others affected by the study	Schools, communities
Sensitivity results	Not undertaken
Heterogeneity	Not explicitly mentioned
Limitations	The smaller size of the intervention schools compared to comparison schools may have inflated the intervention effect.
Funding	This study was funded by the Victorian State Government as part of the Go For Your Life Campaign
Conflict of interest	None declared
**6. Health, Exercise, Nutrition for the Really Young (HENRY)**	
**Bryant et al.(2018)** [27]	
Type of publication	Peer-reviewed protocol, including economic component
Publication title	Cluster randomised controlled feasibility study of HENRY: a community-based intervention aimed at reducing obesity rates in preschool children
Study aim	To assess the feasibility of recruiting local authorities, centres and parents; test processes and time required to train and certify intervention staff; explore HENRY commissioning processes; identify potential sources (and associated impact) of contamination; and consider the feasibility of trial procedures.
Country	United Kingdom
Setting	Children's centres, across two UK local authorities
Design	Within trial cost analysis
Perspective	Not explicitly mentioned

Target population	Parents of the children of children’s centres
Sample size	120 parents from the participating children’s centres
Intervention	HENRY is an 8-week programme delivered in Children's Centres, aiming to provide parents with skills and knowledge to support healthy lifestyles in preschool children and their families. It is currently delivered in ~ 35 local areas across England and Wales by trained health and community practitioners. Training includes:<br>Centre Level Training: to equip staff with knowledge and skills to promote and provide healthy nutrition within early years settings and support parents to provide healthy family lifestyles and nutrition for their families. The theoretical underpinning combines proven models of behaviour change including the Family Partnership Model, motivational interviewing and solution-focused support.<br>Practitioner Level Training: to deliver the 8-week HENRY programme, which aims to build parents' skills, knowledge and confidence to change old habits and provide healthier nutrition for their young children.
Comparator	Children's Centres within local authorities that have been randomised to the control arm will continue with usual practice. Parents attending another programme (Stay and Play) will be invited to take part in the research. At the end of the follow-up period, they will be offered training to deliver HENRY programmes although this will not be compulsory.
Time horizon	One year
Currency, price year and conversion	Not explicitly mentioned
Discount rate	Not explicitly mentioned
Measurement and valuation of resources and costs	Health care resource use data for the child’s health and the parent within the NHS (health services, hospital, social services) as well as time off work in relation with HENRY. A study-specific health resource use questionnaire will<br>be used to collect information on primary and secondary health care utilisation for both the parents and the child at follow-up. Study will collect out of pocket expenses such as parents’ expenses in relation with travel for the intervention and other private expenses (e.g. extra food expenses, extra activities) to improve child’s diet. Study will also collect parents’ lost productivity (time off work) in relation to child’s health and attendance to the programme.<br>Wherever possible, unit costs for resources will be obtained from national sources such as the British National Formulary [32] and the PSSRU Costs of Health and<br>Social Care [33]. NHS and social service resource use will be identified through direct observation of the treatment provided within the feasibility study and through the structured questionnaire for collection of all other service use.
Cost categories	Out of pocket expenses of parents in relation to intervention<br>• Travel<br>• Extra food expenses<br>• Extra activities<br>• Lost productivity (time off work)
Selection of outcomes	1. Feasibility of the intervention (e.g. recruitment rate, training and quality assurance, acceptability, contamination)<br>2. Child BMI z-score

	3. An incremental cost per increase of parent engagement<br>**Secondary:**<br>1. Primary caregiver BMI<br>2. Family eating/activities<br>3. Parenting self-efficacy<br>4. Feeding<br>5. Dental health
Measurement of outcomes	Feasibility: Recruitment rate will be followed throughout the study period.<br>Training and quality assurance: Number of staff attending training, changes in knowledge and practice before and after training, and programme delivery<br>quality assurance. Variability of gender-adjusted body mass index (BMI) in both arms; difference between arms and 95% confidence intervals; and estimation of clustering effect and cluster size.<br>Child BMI: Age- and gender-adjusted height(m)/weight(kg)2) measured by NatCen interviewers
Measurement and valuation of outcomes	Not applicable
Analytical method	As this is a feasibility study, the analysis will focus on descriptive statistics and confidence interval (CI) estimation rather than formal hypothesis testing. The trial is not powered to provide a precise estimate of the level of clustering relating<br>to group effects, but it will allow an investigation of this effect, which will inform the sample size estimation for the definitive trial. No formal analyses are<br>planned until after the trial is closed to recruitment and follow-up and the required number of local authorities/centres have been randomised and the required number of parents have been registered. Final analysis will be carried out when all available outcome data has been collected. Participant retention during follow-up, including the number of centres/parents withdrawing from the study and the timing of and reasons for the withdrawal will be presented overall, by arm and time-point. Levels of missing self-reported outcome data, both at the individual item level and for entire outcome measures will be reported overall, by time-point and by treatment arm.
Rationale and description of model	Not applicable
Model assumptions	Not applicable
Characterising heterogeneity	Not applicable
Characterising distributional effects	Not explicitly mentioned
Uncertainty analysis	Not applicable
Approach to engagement with patients and others affected by the study	Parents, children, local authorities
Sensitivity analysis	Not discussed
Key findings (study parameters, incremental costs and outcomes)	Not applicable

Sensitivity results	Not applicable
Heterogeneity	Not explicitly mentioned
Limitations	Not explicitly mentioned
Funding	The trial is funded by the NIHR Trainees Coordinating Programme awarded to the chief investigator (MB) (CDF-2014-07-052). The views expressed are those of the authors and not necessarily those of the NHS, the NIHR or the Department of Health.
Conflict of interest	None declared
**7. Healthy Habits Happy Homes (4H) Scotland**	
**Gillespie et al. (2019)** [28]	
Type of publication	Peer-reviewed protocol, including costing protocol
Publication title	Protocol for Healthy Habits Happy Homes (4H) Scotland: feasibility of a participatory approach to adaptation and implementation of a study aimed at early prevention of obesity
Study aim	The study aims to: (1) describe the participatory process and methods utilised in stage 1 and 2 of the 4H logic model, (2) describe elements of co-production and CBPR that were utilised to enable adaptations of the original 4H study and (3) outline how the feasibility and acceptability of 4H Scotland will be tested and evaluated.
Country	Scotland
Setting	North East area of Dundee City
Design	Cost analysis alongside trial
Perspective	Not clearly stated
Target population	Preschool children (2 to 5.5 years)
Sample size	40 participant families with children aged 2-5.5 years
Intervention	Families randomised to the intervention group will receive monthly visits to the home over 6 months plus contact every 2 weeks via SMS. Families will be supported to make positive lifestyle changes towards meeting or exceeding UK guidelines or recommendations linked to four energy balance related behaviours of sleep, physical activity, screen time and family meal routine.
Comparator	The control group will receive general healthy lifestyle information linked to sleep routine, family meals, physical activity and screen time each month mailed or emailed. This information includes materials issued routinely by primary care early years' health workers in Scotland.
Time horizon	Not clearly stated
Currency, price year and conversion	Not explicitly mentioned
Discount rate	Not explicitly mentioned
Measurement and valuation of resources and costs	Cost parameters will be analysed and would be based on those used in an randomised controlled trial of an obesity treatment intervention carried out in Scotland [29]. The study considered the amount of time spent if dietitians, administration costs, traveling for home visits (intervention only) and training costs (intervention only) [29]. No other details of estimation is stated.
Cost categories	• Time cost<br>• Training cost<br>• Travel cost,<br>• Promotion and delivery pf intervention
Selection of outcomes	Primary: acceptability and practicability of 4H Scotland<br>Secondary:

	• Child physical activity, sedentary behaviour and sleep<br>• Child screen time<br>• Family eating meals together<br>• Child BMI z-score<br>• Child health-related quality of life<br>• Child body composition (bioelectrical impedance)
Measurement of outcomes	BMI was calculated using direct measure of child height and weight, and then z-scores against the WHO reference curves. Parent questionnaires: Lunch box survey, 24-h food record; Child-report through child questionnaire of quality of life using the 10-item version of the international self-reported measure of quality of life; School reported audit, Principal exit interviews, Teacher-reported school- and class-based nutrition and physical activity initiatives
Measurement and valuation of outcomes	Not applicable
Analytical method	Cost parameters will be analysed and would be based on those used in a randomised controlled trial of an obesity treatment intervention carried out in Scotland; would include: researcher time in lead up, promotion and delivery of intervention; travel; training.
Rationale and description of model	Not applicable
Model assumptions	Not applicable
Characterising heterogeneity	Not explicitly mentioned
Characterising distributional effects	Not explicitly mentioned
Uncertainty analysis	Not applicable
Approach to engagement with patients and others affected by the study	Parents, children, local authorities
Sensitivity analysis	Not undertaken
Key findings (study parameters, incremental costs and outcomes)	Not applicable
Sensitivity results	Not applicable
Heterogeneity	Not applicable
Limitations	Generalisability is limited due to short duration and a small number of participant families
Funding	This work was supported by The Hannah Dairy Research Foundation (HDRF).
Conflict of interest	None declared
**8. Obesity Prevention and Lifestyle (OPAL)**	
**Bell et al. (2016)** [30]	
Type of publication	Report prepared for the Department for Health and Ageing
Publication title	OPAL EVALUATION PROJECT FINAL REPORT
Study aim	This report summarises the key outcome data for the Flinders OPAL Evaluation Project.
Country	Australia
Setting	Early childhood centres, primary and secondary schools from 20 South Australian communities and one Northern Territory community.
Design	Cost analysis alongside a cross-sectional intervention

Perspective	Not explicitly mentioned
Target population	Grades 4 to 6 (9-11 years old) children
Sample size	For cost analysis: 282,820 beneficiaries were considered
Intervention	OPAL enables healthy eating and physical activity to come to life in each community through a range of themes.<br>Theme 1: 'Water. The Original Cool Drink': Encouraging the replacement of<br>sweet drinks with water.<br>Theme 2: 'Give the screen a rest. Active play is best': Encouraging less screen time in favour of outdoor activity.<br>Theme 3: 'Make it a fresh snack': Encouraging the replacement of 'junk' food snacks with healthy options.<br>Theme 4: 'Think Feet First - step, cycle, scoot to school': Encouraging children and families to leave the car at home and actively travel to and from school.<br>Theme 5: 'A Health Brekky is easy as Peel, Pour, Pop': Promoting a healthy breakfast.<br>Theme 6: 'Life looks brighter outside': Promoting families to be active in local parks and playgrounds.
Comparator	Current practice
Time horizon	3-year (intervention cost only)
Currency, price year and conversion	AUD, price year and conversion were not clearly stated
Discount rate	Not explicitly mentioned
Measurement and valuation of resources and costs	The total costs relating to the provision of the OPAL program reflect two main elements: firstly, the costs associated with the development and administration of the State Co-ordination Unit and secondly, the costs associated with council expenditures. The costs associated with Local Government Council expenditures include grants and expenditures include grants made by SA health to local councils plus the additional local council expenditures attributed to the OPAL program. In the absence of complete information from Local Councils, additional expenditures have been calculated based on the initial OPAL agreement that SA health funded grants would be matched one on one by local government funding. The total costs relating to the provision of the OPAL program (state wide coordination unit, research and evaluation, grants to local councils and additional local council expenditures) were calculated and divided by the number of individuals in each of the intervention communities in phases 1 and 2 who could reasonably have been expected to have benefited from the OPAL programme to estimate the average total cost of the OPAL program at an individual level.<br>Further examination of the single platform data revealed that 100% of the total OPAL program expenditures could be attributed to activities relating to children in the 0-18 year old age range. This information was utilised to generate the average costs of the OPAL program at the individual level for students in the intervention communities.
Cost categories	The costs associated with the State Co-ordination Unit in phases 1 and 2<br>• Coordination (State Co-ordination Unit and license agreements)<br>• Administration (stationary and the development of a single IT platform)<br>• Awareness and social marketing

	• Education (staff training and development, travel (national and international).<br>• Salaries (OPAL field staff including equivalent FTE time).<br>The costs associated with Local Government Council expenditures<br>• Grants made by SA health to local councils plus the additional local council expenditures<br>• Additional expenditures
Selection of outcomes	• Children's weight status<br>• QALYs
Measurement of outcomes	Anthropometric measures (The guidelines are based on the protocols of the International Society for the Advancement of Kinanthropometry).<br>All measurements were obscured from the child's view and recorded by the researcher without being discussed with the child, in line with the Body Image<br>Guidelines developed and endorsed by the OPAL Scientific Advisory Committee.<br>Height, weight and waist circumference were determined as the mean of two measures or the median if three measures were taken. Body Mass Index (BMI) was calculated as weight (kg) divided by height (m) squared. Children were categorised as underweight, normal weight, overweight or obese using the International Obesity Taskforce cut-points [31, 32], as per that for 4-5 year growth data. Similarly, BMIs were converted to age- and sex-specific z-scores using the UK 1990 reference data. [33] Means (height, weight, waist circumference, BMI, BMI z-score) or proportions (prevalence of underweight, healthy weight, overweight, obesity, and combined overweight and obesity) are reported as cross-sectional data for baseline (year 3) and final (year 5) and the changes across the two years are reported and analysed for statistical significance.
Measurement and valuation of outcomes	The CHU9D instrument was administered as a component of the OPAL evaluation. The instrument formed part of the child survey for primary school students. Participants were instructed to complete the CHU9D from the perspective of their own current health. [34] The instrument was scored using the newly developed Australian adolescent specific scoring algorithm [35]. The algorithm is preference based, generating utility values on the 0 to 1 QALY scale, and is thereby suitable for application in the measurement and valuation of health benefits for the economic evaluation of the OPAL program.
Analytical method	The data were collected, checked and edited before being analysed. All statistical analyses were conducted using IBM SPSS Statistics version 22 (SPSS Inc., Chicago, IL, USA), STATA statistical software, version 14.0 (StataCorp 2012), and R version 3.1.2 (R Core Team 2014). Means were calculated for continuous data. Proportions are presented as percentages of the respective denominator.<br>A linear regression model was used to estimate the changes of continuous measures and binary logistic model was used to estimate the relative change of binary measures (proportions) between year 3 (baseline) and year 5 (final) for intervention and comparison communities. A multilevel modelling approach was adopted as a result of the hierarchical structure of the data (children nested in schools). This model was used to explore changes from baseline to final across

	intervention and comparison communities in weight status, diet, PA, SB, active travel, neighbourhood environments, and food security.<br>All analyses were performed with two-tailed tests and the level of significance<br>was set at P < 0.05. Where appropriate, 95% CIs were reported along with P values
Rationale and description of model	Not applicable
Model assumptions	Not applicable
Characterising heterogeneity	Not explicitly mentioned
Characterising distributional effects	Not explicitly mentioned
Uncertainty analysis	Not applicable
Approach to engagement with patients and others affected by the study	Schools, communities
Sensitivity analysis	Not undertaken
Key findings (study parameters, incremental costs and outcomes)	**Effectiveness**<br>**Phase 1**<br>• There was a 0.05 point decrease in BMI, and 0.04 point decrease in BMI z-score, from baseline to final in intervention, in comparison to a 0.07 point increase in BMI and 0.06 point increase in BMI z score in comparison.<br>Differences were not statistically significant, (BMI -0.12, 95%CI -0.35 to 0.11, p=0.295; BMI z-score -0.10, 95%CI -0.25 to 0.05, p=0.181).<br>• Using the multilevel model, there was a non-statistically significant 0.25 point decrease (-0.25, 95%CI: 2.36 to 1.86, p=0.815) in BMI, and non-statistically significant 0.04 point decrease (-0.04, 95%CI -0.12 to 0.04), p=0.306) in BMI z-score, from baseline to final in intervention. In comparison, there was a nonstatistically significant increase in BMI (2.64, 95%CI -0.08 – 5.37, p=0.057) and BMI z-score (0.06, 95%CI 6.32 – 0.53, p=0.205) from baseline to final in COMP.<br>• There was a statistically significant difference in age between baseline and final by -0.08 years (95%CI -0.12 - -0.04, p<0.001) in INT and -0.09 (95%CI -0.13 - -0.04, p=0.001) in COMP.<br>**Phase 2**<br>• There were no statistically significant changes in BMI z-score over time in INT or COMP, or any statistically significant differences in change over time between groups.<br>• Using the multilevel model, there was a non-statistically significant increase in BMI (0.10, 95%CI -0.06 – 0.26, p=0.237) and BMI z-score (0.06, 95%CI -0.04 – 0.15, p=0.219) in INT and a non-statistically significant decreased in BMI (-0.02, 95%CI -0.19 – 0.15, p=) and BMI z-score (-0.03, 95%CI -0.13 – 0.07, p=0.521) in COMP.<br><br>**Cost**<br>**Phase 1 and 2 (between 2008 and 2015)**<br>• The estimated total costs: $19,384,258<br>• The average total cost per person: $68.54 (282,820 beneficiaries)

	• Children 0-18 year old: the average cost: $287.93 per child (67,322 beneficiaries)
Sensitivity results	Not undertaken
Heterogeneity	To account for the heterogeneity of measures between children within the community, the models were adjusted by a small unit of area called suburb.
Limitations	• Cross-sectional nature of the baseline and follow up populations for the assessment of HRQoL for the intervention and control communities.<br>• CUA focuses on health outcomes and these are typically measured using QALY framework. It was not possible to capture outcomes beyond health in the standard QALY framework in this evaluation<br>**The economic evaluation of public health interventions raises several key methodological challenges:**<br>[1] In contrast to many health care interventions, it is very difficult to conduct a randomised controlled trial of a public health intervention and often other, more pragmatic study designs are needed. A related issue is that outcomes for many health care programmes are often adequately captured in the short term whereas public health programmes, in particular prevention programmes, may have long term health impacts.<br><br>[2] Outcomes beyond health may be attributable to public health interventions including reassurance and the creation of an informed public as well as other non-health related outcomes such as education. CUA focuses on health outcomes and these are typically measured using the quality adjusted life years (QALY) framework. Currently, it is not possible to capture outcomes beyond health in the standard QALY framework.<br><br>[3] Equity considerations take on particular importance in the public health sector because reducing inequalities in health (as opposed to reducing inequalities of access to health care treatments and services) is a primary goal of many public health interventions. As such, the equity impacts tend to be much more important for public health interventions where in many cases the main objective of the intervention is to reduce health inequalities.
Funding	SA health
Conflict of interest	NA
**9. Optimising Family Engagement in HENRY (OFTEN)**	
**Bryant et al.(2017)** [36]	
Type of publication	Peer-reviewed protocol, including economic evaluation protocol
Publication title	Effectiveness of an implementation optimisation intervention aimed at increasing parent engagement in HENRY, a childhood obesity prevention programme - the Optimising Family Engagement in HENRY (OFTEN) trial: study protocol for a randomised controlled trial
Study aim	To evaluate the effectiveness of this optimisation intervention in regard to the engagement of parents and cost-effectiveness
Country	United Kingdom
Setting	Children's centres, across two UK local authorities
Design	Trial-based CEA
Perspective	Not explicitly mentioned
Target population	Parents of the children of children's centres
Sample size	24 local authorities, 144 children's centres

Intervention	Parents in the intervention arm attended the 8-week HENRY (Health, Exercise, Nutrition for the Really Young) programme delivered in children centre's to<br>groups of 8–10 parents. It aims to provide parents with the skills and knowledge to support a healthy lifestyle in preschool children and their families.<br>Training for intervention delivery is split into two stages:<br>Centre level training: equipping staff with skills and knowledge to promote and provide healthy nutrition in early years settings and to support parents to provide healthy lifestyle and nutrition for their families; and (2) Practitioner level training to deliver HENRY programme to families: training staff to deliver the 8 week programme. Both types of training are underpinned by a combination of proven models of behaviour change, including the Family Partnership Model, motivational interviewing and solution focused support. Topics covered in the HENRY programme include eating habits, balancing healthy meals and snacks, child appropriate portion sizes, emotional wellbeing, parenting skills and activity. Services provided by children's centres that were deemed similar to those of HENRY (e.g. parenting, healthy eating), as well as services attended by study participants, were recorded.
Comparator	Standard care was continued in centres assigned to the control condition. These centres delivered all their usual programmes (including programmes such as 'stay and play', 'cook and eat', baby massage and other parenting courses). Staff did not receive HENRY training or materials. Following all data collection, centres in the control group received free HENRY training to enable delivery of the HENRY sessions. Parents in control centres were offered attendance at HENRY once the data collection for the study had ended (i.e. a waiting list). All services similar to those of the HENRY programme that were provided by the centres were recorded (both in the control centres and those allocated to HENRY).
Time horizon	One year
Currency, price year and conversion	Not clearly stated
Discount rate	Not clearly stated
Measurement and valuation of resources and costs	The study will include the costs required to deliver the intervention (gathered and transferred from HENRY) and the routinely collected outcome data.
Cost categories	Costs required to deliver the intervention (from project record)
Selection of outcomes	1. The proportion of centres enrolling at least eight parents per programme and<br>2. The proportion of centres with at least 75% of parents attending five of eight sessions per programme.<br>3. An incremental cost per increase of parent engagement<br>**Secondary:**<br>1. Proportion of centres achieving all targets for enrolment, attrition and parent compliance<br>2. Parent adherence to HENRY programme content<br>3. Impact of HENRY on parenting and family health

Measurement of outcomes	Parent adherence : defined as the proportion of parents reporting an increase of 0.5 in the daily frequency of consumption of fruits and vegetables by children per programme<br>Impact of HENRY: assessed by parent-report, including the following (Parenting self-efficacy, Eating behaviours, Family activity, Child screen time, Intake of key indicator foods per day,
Measurement and valuation of outcomes	Not applicable
Analytical method	The study will model the benefits of optimisation intervention over the costs of implementation and will seek commissioners' willingness to pay for this.<br><br>• Will conduct a meeting with commissioners to determine their willingness to pay per additional unit of effectiveness (parent engagement) before concluding<br>whether the optimisation intervention can be considered cost-effective.<br>• Contingent valuation techniques and design an experiment will be used to estimate the point at which commissioners consider that the costs of the programme are acceptably offset by the benefits of engaging effectively with parents.<br>• Cost-effectiveness analysis will include trial endpoints (including parent enrolment and attrition rates) and costs. Cost-effectiveness acceptability curves will describe the probability that the optimisation intervention is cost-effective for a range of maximum monetary values that a decision-maker might be willing to pay for a particular unit change in outcome.<br>• Statistical analyses will be carried out using ITT principle, and statistical significance will be assessed at the 5% significance level with 95% CIs
Rationale and description of model	Not applicable
Model assumptions	Not applicable
Characterising heterogeneity	Not applicable
Characterising distributional effects	Not explicitly mentioned
Uncertainty analysis	Not applicable
Approach to engagement with patients and others affected by the study	Parents, children, communities.
Sensitivity analysis	Not discussed
Key findings (study parameters, incremental costs and outcomes)	Not applicable
Sensitivity results	Not applicable
Heterogeneity	Not explicitly mentioned
Limitations	Not explicitly mentioned
Funding	The trial is funded by the NIHR Trainees Coordinating Programme awarded to the chief investigator (MB) (CDF-2014-07-052). The views expressed are those of the authors and not necessarily those of the NHS, the NIHR or the Department of Health.
Conflict of interest	None declared
**10. Pacific OPIC Project (Obesity Prevention In Communities)**	

Swinburn et al. (2007) [37]; Swinburn et al. (2011) [38]	
Type of publication	Peer-reviewed protocols, including economic evaluation protocol
Publication title	The Pacific OPIC Project (Obesity Prevention In Communities) – Objectives and designs; The Pacific Obesity Prevention in Communities project: project overview and methods
Study aim	The studies aim to:<br>1) Determine the overall impact of comprehensive, community-based intervention programs on overweight/obesity prevalence in youth;<br>2) Assess the feasibility of the specific intervention components and their impacts on eating and physical activity patterns;<br>3) Understand the socio-cultural factors that promote obesity and how they can be influenced;<br>4) Identify the effects of food-related policies in Fiji and Tonga and how they might be changed;<br>5) Estimate the overall burden of childhood obesity (including loss of quality of life);<br>6) Determine the cost-effectiveness of the four intervention programmes in order to inform decisions about the optimal allocation of resources for obesity prevention., and;<br>7) Increase the capacity for obesity prevention research and action in Pacifi c populations
Country	Fiji, Tonga, New Zealand, Australia
Setting	Australia: Secondary schools in the East Geelong/Bellarine and South-Western region of Victoria<br>Fiji: The peri-urban area of Nasinu and west-side of Viti Levu main island<br>Tonga: The main island of Tongatapu and island of Vava'u<br>New Zealand: South Auckland
Design	Trial based CUA
Perspective	Not explicitly mentioned
Target population	Adolescents (12-18 years)
Sample size	Australia: Five intervention and seven comparison schools<br>Fiji: Seven intervention and eleven comparison schools<br>Tonga: All students from 3 districts of Tongatapu as intervention and all students from Vava's island as comparison.<br>NewZealand: Four intervention and two comparison schools
Intervention	Analysis Grids for Elements Related to Obesity workshops with stakeholders comprised a guided process by which communities developed agreed action plans to address priority obesogenic behaviours and build community capacity to promote healthy eating and physical activity. While the interventions that arose from the action plans varied somewhat across sites, there were many commonalities such as targeting reductions in sweet drink and energy-dense snack consumption and increasing structured and unstructured physical activity.<br>The intervention targeted an increase in community capacity which was envisaged to influence local environments and individual knowledge and attitudes. These in turn were postulated to influence diet and physical activity patterns and changes in BMI and quality of life which could then be modelled to changes in quality-adjusted life years gained. Variables such as gender, ethnicity and age were considered moderators in that they could have interactive effect with the intervention producing differential impacts on the outcome variables.

Comparator	Current practice
Time horizon	Five-years (3-year intervention)
Currency, price year and conversion	Local currencies, 2005
Discount rate	Not explicitly mentioned
Measurement and valuation of resources and costs	The intervention costing (over three years) involves a large number of players, and has required onsite training and flexible data collection methods. Resource use associated with all intervention activities is documented through a diary approach, and access to records such as invoices, minutes of meetings, staff notes etc. Current practice as reflected by obesity prevention activities in the comparator schools is also being costed.
Cost categories	Intervention related expenditure categories (No further details)
Selection of outcomes	• Change in BMI or BMI-z score<br>• Knowledge (indicator questions)<br>• Quality of life (PedsQoL, AQoL2)<br>• Perceptions (body size, role models at home and school)<br>• Environments (school audit)<br>• QALYs
Measurement of outcomes	• Anthropometry (height, weight, waist)<br>• Body composition (bioelectrical impedance)<br>• Behaviours were assessed through questionnaire using standard Questions where possible. Audit tool used for school environments, supplemented by youth responses to role model questions. Collected at baseline and follow-up.
Measurement and valuation of outcomes	Two quality of life instruments are being administered at baseline and follow-up to facilitate description of the health burden of adolescent overweight and obesity and as an outcome measure in a cost-utility analysis of the interventions. The latter will enable a comparison of the efficiency of obesity prevention with a broader range of health care interventions. As the AQoL-2 (Assessment of Quality of Life) was developed for Australian adults, modification and cultural validation was done for adolescent use (through onsite focus groups) with recalibration of the utility weights. The latter required the completion of ten 'time trade-off' scenarios by samples of 60 adolescents in each site, conducted on a small group basis in the intervention schools.
Analytical method	In the final component of the economic studies, the cost and outcome datasets were compiled with local data on the prevalence of obesity-related diseases and their costs in an economic model to describe the disease burden and healthcare cost implications of adolescent obesity. This model can also be used to predict the costs and benefits downstream as a result of the interventions through their capacity to reduce obesity-related disease.<br>No detail method for cost-effectiveness analysis was described. 'DALYs saved' specified as outcome measurement with no further detail.
Rationale and description of model	Not applicable
Model assumptions	Not applicable
Characterising heterogeneity	Not explicitly mentioned

Characterising distributional effects	Not explicitly mentioned
Uncertainty analysis	Not applicable
Approach to engagement with patients and others affected by the study	Schools, communities
Sensitivity analysis	Not explicitly mentioned
Key findings (study parameters, incremental costs and outcomes)	Not applicable
Sensitivity results	Not applicable
Heterogeneity	Not explicitly mentioned
Limitations	Quasi-experimental design (cRCT would have technical)<br>CUA mentioned: data collection on cost and QoL explained.
Funding	Wellcome Trust (UK) (grant reference number 071637/Z/ 03/Z), the National Health and Medical Research Council (Australia) and the Health Research Council (New Zealand)
Conflict of interest	Not specified
**11.The Romp & Chomp early childhood obesity prevention intervention**	
**Tran et al. (2022)[39]**	
Type of publication	Peer-reviewed economic evaluation
Publication title	Cost-effectiveness of scaling up a whole-of-community intervention: the Romp & Chomp early childhood obesity prevention intervention
Study aim	The study assessed the cost-effectiveness of the Romp & Chomp community-wide early childhood obesity prevention intervention if delivered across Australia compared to no intervention.
Country	Australia
Setting	City of Greater Geelong (CoGG) and the Borough of Queenscliffe (BoQ) in Victoria, Australia
Design	Modelled CEA & CUA
Perspective	Funder
Target population	Australian children from 0 to 5 years of age
Sample size	Australian children aged from 0 to 5 years (n=1,906,075)
Intervention	The intervention was conducted between 2004 and 2008 with an aim to change policy, sociocultural, and physical aspects of early childhood environments to favor obesity prevention.<br>The program's 6 key messages were as follows: 1) limit food; 2) move, play and<br>go; 3) turn off, switch to play; 4) tap into water every day; 5) stride and ride; and 6) plant fruit and vegetables in your lunchbox.<br>Romp & Chomp targeted the entire population of children aged 0–5 y operating primarily through 8 Long Day Care centers, 76 Family Day Care service, 45 preschools, 24 Maternal Child Health Service centres, regional<br>immunization services, and community health services.<br>The aim of Romp & Chomp was to increase the capacity of the CoGG and the BoQ (the intervention site) to promote healthy eating and active play and to achieve healthy weight in children,5 y of age. The Romp&Chomp action plan was

	developed with extensive community consultation and stakeholder engagement, and a management committee of stakeholders oversaw its implementation.
Comparator	No intervention (a nationally representative cohort of Australian children that did not receive the R&C intervention)
Time horizon	10-year
Currency, price year and conversion	AUD, 2018
Discount rate	5% for both costs and benefits
Measurement and valuation of resources and costs	Costs were estimated retrospectively using trial records and micro-costing techniques. All assumptions on how the intervention would be implemented at scale were based on the existing literature on community-wide obesity prevention intervention [40], the management structure reported in trial records (unpublished documents) and in consultation with members of the R&C research team.
Cost categories	• Time costs - published wage rates including salary on-costs (i.e. overhead costs, superannuation, employer taxes, compensation, and leave loading)<br>• Travel costs- All travel costs to provide intervention training and to promote intervention messages (Health Promotion Officers). Costs estimated using published guidelines.<br>• Intervention material costs - Marketing and promotional materials (e.g., toolkit documents, display posters, fact sheets, and stickers).
Selection of outcomes	• Incremental cost-effectiveness ratios (ICERs)<br>• Cost per BMI unit avoided<br>• Cost per quality-adjusted life year (QALY) gained
Measurement of outcomes	Intervention effect was estimated using a repeat cross-sectional quasi-experimental design to measure the differences in outcomes between the population exposed to the R&C intervention (pre- and post-intervention data collection). The intervention effect sizes were the average weight of all the children in the intervention communities compared to those of the non-intervention communities. The classification of weight status was based on WHO growth standards. [41]
Measurement and valuation of outcomes	QALY weights associated with child weight status to inform the estimation of QALYs were obtained from a recent systematic review and meta-analysis. [42]
Analytical method	All analyses were conducted in Stata version 16.1. [43]
Rationale and description of model	Estimation of cost-effectiveness of community-wide interventions in early childhood populations across shorter time horizons is limited.[44] Therefore, such evidence is important because it could assist decision making in a shorter-term, policy-relevant timeframe.[45]<br>A deterministic micro-simulation model (the EPOCH model) [46] was used to predict individual level child BMI trajectories, weight status and associated QALYs and healthcare costs from age 4 to 15 years, extrapolating the trial-based intervention effects nationally to children in the target age group. Simulated BMI and QALYs to age 15 years were modelled using trial data at age 3.5 years. Each child was set on a different BMI trajectory based on the different starting BMI measured at the end of the trial. [46, 47]

Model assumptions	• Each child was set on a different BMI trajectory based on the different starting BMI measured at the end of the trial [46, 47]. Modelled CEA estimated the incremental cost per BMI unit avoided (AUD/BMI unit avoided) at age 15 years<br>• Intervention effect size (-0.06kg/m^2) was applied to child BMI at age 4/5 years to estimate BMI trajectories of the intervention group to age 14/15 years and compared to the trajectories of the same cohort without any intervention effects applied.<br>• The QALY weights of children with healthy weight, overweight and obesity were 0.85, 0.83 and 0.82 respectively.[42] Healthcare costs of participants to age 15 years were modelled following a 'top down' method, using administrative records of annual hospital, doctor and medical costs by age adjusted by weight status.
Characterising heterogeneity	Base case assumptions included the adoption of electronic intervention resources given the heterogeneity in Early childhood education settings between Australian states and territories.
Characterising distributional effects	Not explicitly mentioned
Uncertainty analysis	The joint uncertainty around costs and QALYs was estimated by creating 1,000 bootstrapped samples of simulated data that was used to calculate the probability of the intervention being cost-effective compared to the comparator at different willingness to pay thresholds.[47]
Approach to engagement with patients and others affected by the study	Family day care centres, centre-based long day care centres, preschools
Sensitivity analysis	A series of univariate and multivariate sensitivity analyses were performed to evaluate the impact of assumptions (varying intervention cost for children aged 4 to 5 years (S1), high cost categories (S2), interventions effects with worst case (S3), and discount rate to 3% (S4)).
Key findings (study parameters, incremental costs and outcomes)	**Cost**<br>• Total intervention cost per year: AUD177,536,705<br>• Mean intervention cost per participant: AUD93<br>• Mean healthcare cost saving per participant: AUD15<br>• Incremental total cost (95% CI): AUD78 ($54;$103)<br>**Effectiveness**<br>• Mean BMI unit avoided (95% CI): 0.07 (-0.01; 0.16)<br>**Cost-effectiveness**<br>• Mean ICER, AUD per BMI unit avoided (95% CI): AUD1,126 (AUD 5,958)-Dominated<br>• Mean ICER, AUD per QALY gained (95% CI): AUD 26,399 (AUD246,826)-Dominated<br>• Probability of being cost-effective 64%
Sensitivity results	**Mean intervention cost per participant**<br>S1: children 4-5 years: AUD276<br>S2: High intervention cost estimate: AUD276<br>S3: Worst case: AUD475<br>S4: 3% discount rate: AUD95<br>**Incremental total cost (95% CIs)**<br>S1: Children 4-5 years: AUD261 ($234; $286)<br>S2: High intervention cost estimate: AUD145 ($121; $170)<br>S3: Worst case: AUD472 ($450; $497)<br>S4: 3% discount rate: AUD78 ($48; $109)<br>**Mean BMI unit avoided (95% CI) at age 15 years**

	S3: Worst case: 0.01 (-0.07; 0.08)<br>S4: 3% discount rate: 0.09 (-0.00; 0.19)<br>**Mean ICER, AUD per BMI unit avoided (95% CI)**<br>S1: Children 4-5 years: AUD3,767 ($17,683)-Dominated<br>S2: High intervention cost estimate: AUD2,089 ($9,939)-Dominated<br>S3: Worst case: AUD40,719 ($173,331)-Dominated<br>S4: 3% discount rate: AUD871 ($6,035)-Dominated<br>**Mean ICER per QALY gained (95% CI)**<br>S1: Children 4-5 years: AUD88,332 ($875,591)-Dominated<br>S2: High intervention cost estimate: AUD48,974 ($481,265)-Dominated<br>S3: Worst case: AUD956,146 ($1,373,912)-Dominated<br>S4: 3% discount rate: AUD22,894 ($182,830)-Dominated<br>**Probability of being cost-effective**<br>S1: Children 4-5 years: 31% (Not cost-effective)<br>S2: High intervention cost estimate: 53% (Cost-effective)<br>S3: Worst case: 1.6% (Not cost-effective)<br>S4: 3% discount rate: 64% (Cost-effective)
Heterogeneity	Base case assumptions included the adoption of electronic intervention resources given the heterogeneity in Early childhood education and care (ECEC) settings between Australian states and territories and the constantly evolving nature of the ECEC environment.
Limitations	• The assumptions of model required to extrapolate costs and effects nationally, although the analysis followed methodologies in the published literature<br>• No sensitivity analysis conducted using decay of intervention effect<br>• No uncertainty analysis was conducted to test the impacts of the ranges and distributions of input parameters on the results<br>• Long-term health burden associated with high BMI was not estimated
Funding	The National Health and Medical Research Council (NHMRC) funded Centre of Research Excellence in Early Prevention of Obesity in Childhood (APPID1101675)
Conflict of interest	The authors declare that the research was conducted in the absence of any commercial or financial relationships that could be construed as a potential conflict of interest.
**12. Shape Up Somerville (SUS)**	
**Coffield et al. (2019)[48]**	
Type of publication	Peer-reviewed return on investment
Publication title	Shape Up Somerville's return on investment: Multi-group exposure generates net-benefits in a child obesity intervention
Study aim	This study offers insight into the possible return on investment of a nationally recognized whole system approach to reducing and preventing childhood obesity and provides information for communities to use while discussing the practicality and sustainability of community-wide environmental change programs.
Country	USA
Setting	Massachusetts
Design	Trial based CBA
Perspective	A modified societal perspective
Target population	Children grades 1-3
Sample size	For economic evaluation: 1,600 children and 1,453 parents were used to estimate SUS’s benefits.

Intervention	Influence multiple aspects of child’s day at school (before [make free healthier breakfast, walk to school programs], during [change school lunch environment, classroom curriculum, make recess more active], and after school [curriculum, walk from school campaign]). Home environment targeted through parent outreach and education (e.g. newsletters, nutrition forums, family events). Restaurant initiative in community, training local physicians on approaching and counselling families with an overweight or obese child; regular local media placement; development of community-wide policies (including comprehensive Wellness Policy).
Comparator	Control communities with no intervention
Time horizon	10-year
Currency, price year and conversion	USD, 2014
Discount rate	3% for costs and benefits
Measurement and valuation of resources and costs	Program costs included SUS's non-research based costs for the 2003/04 and 2004/05 school years Program. Cost was estimated directly from SUS documents for the first study year. Year two program cost estimates were projected from year one costs and events that occurred during the second study year.<br>Labor costs included community and parent outreach, professional development, substitute teachers, newsletters/material preparation, on-site consultation, and other such items. Equipment costs include items such as an oven, other kitchen equipment, and SUS curriculum. Equipment was annuitized over 10-years assuming a $0.00 scrap value. Equipment cost represents two-years of this annuitized value. Material cost include items such as food donations, incentives, office supplies, printing, sport equipment, material for parent forums, and clothing. Facility costs include office space and utilities. The cost of office space was based upon Boston, Massachusetts market rates while utility costs were based upon utility rates for New England.<br>Cost excluded: Costs associated with evaluating SUS, participant time required to engage in SUS.
Cost categories	• Labor cost (e.g., trainings, community outreach)<br>• Capital equipment (e.g.. food service equipment)<br>• Material cost (e.g., SUS banner for the family)<br>• Facility costs (e.g. office space and utilities).
Selection of outcomes	Net-benefit
Measurement of outcomes	SUS's benefits were projected for each time horizon year for children and parents separately using population level data. These separate, annual calculations adjusted for projected changes in SUS's exposed population size and depreciation in SUS's treatment effect over the 10-year time horizon. SUS's total benefit was the sum of the 10-annual child and the 10-annual parent benefit calculations. The annual benefit calculation for children was the product of a population-wide<br>treatment effect and the healthcare costs associated with a one-point BMI z-score change.<br>To estimate the healthcare costs associated with BMI z-score (children) and BMI (parents) changes, data were pooled from the 2008–2013 cross-sectional Medical Expenditure Panel Surveys (MEPS).

Measurement and valuation of outcomes	Not applicable
Analytical method	• The annual benefit calculation for children was the product of a population-wide treatment effect and the healthcare costs associated with a one-point BMI z-score change.<br>• The annual calculation for parents was the product of a population-wide treatment effect and the sum of the healthcare costs and productivity changes associated with a one-point BMI change<br>• A two-part regression model was estimated for each sample to calculate<br>the healthcare costs attributable to a one-point BMI z-score (children) or BMI (parent) change while controlling for socioeconomic and demographic covariates. If a significant association (ρ≤0.05) was not present in a model, the healthcare cost averted for that year was $0.00.<br>• Possible productivity losses averted due to SUS were estimated annually for parents; the estimate, for each time horizon year, was the product of: a) number of sickness-related missed workdays associated with a 1-point BMI change, b) parent population-wide SUS treatment effect, and c) median daily wage of the MEPS sample which was estimated with SAS software version 9.4.<br>• US's costs and benefits were estimated through a return on investment ratio (ROI) calculated by net benefit divided by program costs
Rationale and description of model	Not applicable
Model assumptions	Not applicable
Characterising heterogeneity	Not explicitly mentioned
Characterising distributional effects	Not explicitly mentioned
Uncertainty analysis	Not applicable
Approach to engagement with patients and others affected by the study	Families, communities
Sensitivity analysis	• Estimates were calculated using treatment effect size reductions of 0% to 10% in one-unit increments to examine how the annual 2.62% depreciation of SUS's treatment effect size influenced SUS's ROI assuming SUS's treatment effect size completely dissipates in 10-years with a 10% annual reduction.<br>• ROI changes over the time horizon: the lower and upper intervals were based on the 95% confidence intervals from the healthcare costs and productivity-based regressions and the median wage estimates.<br>• The first year of the annual ROI estimates presents the worst case scenario; the scenario where SUS's estimated treatment effect size completely dissipates at the end of the first year. Sensitivity analyses, where parameters varied singularly and jointly, were also conducted using @RISK
Key findings (study parameters, incremental costs and outcomes)	**Effectiveness/Benefits**<br>• A lower BMI z-score was associated with significantly lower healthcare costs: ages 14–15 (year 5 after the program) and 15–16 (year 6). For one

	child age (12–13; year 3) a lower BMI z-score was associated with significantly higher healthcare costs.<br>• Avert healthcare spending and productivity losses by $581,837 over the 10-year time horizon<br><br>**Cost**<br>• Two-year program costs: $384,717 (labor cost is the highest contributor)<br>• Material cost: $95,530; Equipment cost: $25,087; Labor cost: $237,947; Facility cost: $26,153<br><br>**Cost-benefit in terms of ROI**<br>• SUS generated a projected $1.51 in savings for every $1.00 invested in the program i.e estimated 10-year return per dollar of investment was $0.51<br>• Generated benefits of $197,120 more than its program costs over the 10-year time horizon<br>• SUS generated estimated benefits over each year of the time horizon with total benefits exceeding total costs starting in year 7<br>• SUS's estimated benefits exceeded its estimated costs in years 10 and 5 in the lower and upper ROI estimates
Sensitivity results	• SUS's ROI remains positive until 76.20% of SUS's treatment effect size dissipates over the 10-year period resulting in SUS's annual treatment effect size breakeven depreciation rate of 7.62%.<br>• In the sensitivity analysis, SUS returned a positive ROI in 90.0% of 5000 multivariate iterations with an average ROI of $0.19 (95% uncertainty interval: -$0.09, $0.50).
Heterogeneity	Not explicitly mentioned
Limitations	• Absence of actual year two program cost data<br>• Opportunity cost was not estimated<br>• Excluded any benefits from possible quality of life changes<br>• Some costs incurred by community members not incorporated<br>• Cross-sectional dataset used as US population representation (instead of longitudinal)<br>• these results may produce conservative estimates relative to more innovative modelling methods that account for the indigeneity of BMI and omitted variables
Funding	Major funding for this research was provided by grant R06/CCR121519-01 from the Centers for Disease Control and Prevention (CDC), Atlanta, GA
Conflict of interest	Cawley has personal fees outside of this study from Novo Nordisk and grants from the Robert Wood Johnson Foundation. Collins and Economos have a patent curriculum with royalties paid associated with Shape Up Somerville. Carlson, Coffield, Lee, and Nihiser reported no conflict of interest.
**13. Whole of Systems Trial of Prevention Strategies for Childhood Obesity: WHO STOPS Childhood Obesity**	
**Allender et al. (2016) [49]; Sweeney et al. (2018) [50]**	
Type of publication	Peer-reviewed economic evaluation protocol
Publication title	Whole of Systems Trial of Prevention Strategies for Childhood Obesity: WHO STOPS Childhood Obesity[49]; Protocol for an economic evaluation of WHO STOPS childhood obesity stepped-wedge cluster randomised controlled trial[50].

Study aim	This papers described protocols for WHO STOPS trial to measure the impact of increased action on risk factors for childhood obesity, and the planned economic evaluation of WHO STOPS and examines the methodological challenges for economic evaluation of a complex systems-based intervention.
Country	Australia
Setting	South Western regional and rural communities in Victoria, Australia
Design	Within trial and modelled CUA
Perspective	Funder/organiser and societal perspectives
Target population	Children aged 8-12 years
Sample size	Whole of the community of the respective settings<br>(Estimated sample for project: n=5,050 from 10 included communities)
Intervention	The intervention has three main components<br>**Community engagement and facilitation (component 1)**<br>Community leaders (CLD) will engage in at least two group model building (GMB) sessions. All community members are invited to participate in a whole of community session (or sessions), where participants will (1) review the CLD, (2) identify points across the CLD where community-led actions to reduce obesity-related risk factors can be designed and implemented and (3) form community action groups to take ownership of these proposed actions.<br>**Backbone organisation (component 2)**<br>The WHO STOPS process uses a collective impact approach, which seeks to identify and support a local 'backbone' organisation that will take significant responsibility for fostering, supporting and monitoring the community-led actions (described in component 3). During a pilot testing phase in a proximal, comparable town, the backbone organisation was a locally based public health organisation that allocated personnel time to these tasks.<br>**Community-led actions (component 3)**<br>Component 3 consists of the planning and implementation by community members, of the suite of actions identified in their whole of community sessions, as well as any actions subsequently taken in the community that were motivated by component 1. The aim will be to undertake numerous actions across multiple points in the community, thereby addressing a range of obesity-related risk factors. Actions might be led by community organisations (including local government, health services, schools and sporting clubs), businesses, community groups or individuals.
Comparator	Current practice will be observed in control (step 2) communities. This will include any local strategies targeting obesity-related risk factors implemented at the community level. Current practice itself is dynamic as communities introduce new and phase-out old local strategies. Implementation of the intervention in control communities will be completed 2 years after implementation in intervention (step 1) communities.
Time horizon	Lifetime (100 years)
Currency, price year and conversion	AUD, inflation adjustment will be done at the time of analysis.
Discount rate	Discount rate: 5% in base case, annual rates 3.5% and 0% for sensitivity analysis.

Measurement and valuation of resources and costs	For identification and measurement of costs, study will use a community’s backbone organisation to track community actions and collect data on related resource use for given activities. For each of the components, data will be collected annually or every 2 years or over ongoing activities depending on the actions using different data sources (e.g. project administrative records, meeting attendance sheets). The time contributions of individuals will be costed using opportunity cost principles. Resource use of non-health sector goods and services will be valued at market prices and be informed by best available evidence from Australian-based studies. Where relevant, health resources will be costed as per the Manual of Resource Items for use in submissions to the Commonwealth of Australia's Pharmaceutical Benefits Advisory Committee.
Cost categories	Committee Component 1 & 2:<br>• Personnel time, travel costs, equipment (scales, tablets, etc);<br>GMBs and whole of community sessions.<br>Costs included: personnel time (facilitators and participants), travel, venue/catering, printing/stationery, STICK-E software licence.<br>Meeting facilitation and travel costs. Communication costs including printing and dissemination of newsletters and advertising of meetings and activities.<br>Component 3 (community-led actions)<br>• Personnel time<br>• Venue hire, and equipment<br>• Infrastructure investment
Selection of outcomes	• Change in childhood BMI-z and obesity prevalence<br>• Change in children’s diet and physical activity (PA) behaviours<br>• Change in Quality of life and settings environments for food and PA<br>• Incremental cost-effectiveness ratios ($A) per BMI unit saved<br>• QALY gained.
Measurement of outcomes	• Body Mass Index-z score, Overweight and obesity prevalence (height & weight)<br>• PA: Minutes per day spent in moderate-to-vigorous physical activity and sedentary behavior; Proportion of participants meeting the national Evaluation System. Accelerometer (sub sample) physical activity guidelines and screen-time recommendations (instrument: Modified Core Indicators and Measures of Youth Health and School Health Action, Planning and Evaluation System Accelerometer (sub sample). Diet Type, frequency: Usual serves of: fruit and vegetable daily, Usual frequency of non-core foods (e.g., take-away food, chips, lollies, chocolate), Usual frequency of sugar-sweetened beverages (Instrument: Modified version of the Simple Dietary Questionnaire).
Measurement and valuation of outcomes	HRQoL data will be collected using the PedsQLTM 4.0 Child Report (8–12 years).<br>Given PedsQL is a non-preference-based 23 item instrument that assesses functioning across physical, emotional, social and school domains, where responses are transformed to a score on a 0–100 scale; higher scores reflect better HRQoL. Given PedsQL is a non-preference-based HRQoL instrument, an algorithm will be developed to enable conversion of PedsQL overall scores of study participants to the preference-based Children’s Health Utility 9 Dimension.

	Specifically, a dataset of around 1,800 Australian children aged between 10 and 12 years will be employed.
Analytical method	The optimal mapping algorithm will be chosen based on a series of econometric techniques using a number of goodness-of-fit measures. This will enable estimation of any resulting QALY gains.
Rationale and description of model	An existing multistate life table Markov model will be used. The model was built in Excel (Microsoft Office 2003) and uses the add-in tool Ersatz (EpiGear, V.1.0) for uncertainty analysis.
Model assumptions	Described in detail in Brown et al, the model estimates (for the 2010 Australian population) the extent to which changes in BMI and PA (independent of BMI) impact on the incidence and associated healthcare costs of osteoarthritis of the knee and hip, breast cancer, colon cancer, endometrial cancer, kidney cancer, ischaemic heart disease, hypertensive heart disease, stroke and type 2 diabetes; all causally related to obesity.
Characterising heterogeneity	To investigate between-community variability, all communities will be encouraged to monitor community actions and resource contributions using the community action register. Prior to finishing the economic evaluation, results from the costing of the two selected intervention communities will be discussed with backbone organisations from all intervention communities. Those backbone organisations from communities not comprehensively costed will be asked to consider those results alongside the actions registered in their own registers, and reflect on the extent to which findings appear consistent with their own communities. If perceived heterogeneity is raised, this will be acknowledged as a limitation in the published economic evaluation results.
Characterising distributional effects	Not explicitly mentioned
Uncertainty analysis	Given uncertainty around the maintenance of community responses and treatment effects beyond the trial period, modelling of future benefits and health cost savings will test a range of plausible assumptions of decayed and maintained treatment effect. These may range from full decay over 5 or 10 years through to 100% maintenance of effect. Modest accentuation of treatment effect will also be modelled.
Approach to engagement with patients and others affected by the study	Families, Communities, range of actors involved.
Sensitivity analysis	Extensive analyses will be undertaken to test the sensitivity of results to plausible variations in all trial based and modelled variables, including assumptions around the maintenance of any observed changes in BMI, PA and fruit and vegetable consumption as well the costs of alternative approaches to GMB facilitation
Key findings (study parameters, incremental costs and outcomes)	Not applicable
Sensitivity results	Not applicable
Heterogeneity	Not applicable
Limitations	Not explicitly mentioned

Funding	This study is supported by an NHMRC Partnership Project titled "Whole of Systems Trial of Prevention Strategies for childhood obesity: WHO STOPS childhood obesity" (APP1114118)
Conflict of interest	None declared

Abbreviations: ACs, Community Activity Coordinators AUD, Australian dollars; BMI, Body mass index;*C, comparator; CEA, cost-effectiveness analysis, CDWs, Community Development Workers; CI, confidence interval CUA, cost-utility analysis; DALY, Disability adjusted life year; HALY, health-adjusted life years; HRQoL, health-related quality of life; HUI, health utility index; I, intervention; ICER, incremental cost-effectiveness ratio; ITT, intention-to-treat; LGA, Local Government Areas; N, sample numbers; NA, not applicable; NS, not stated; NZ, New Zealand; NZD: New Zealand dollar; PA, physical activity; QALY, Quality-adjusted life-year; RCT, randomised controlled trial; ROI, Return on investment USD, SA, sensitivity analysis, United States dollars; WHO, World Health Organization*

1 Moher D, Liberati A, Tetzlaff J, Altman DG. Preferred reporting items for systematic reviews and meta-analyses: the PRISMA statement. *Annals of internal medicine*. 2009; 151: 264-69.
2 Flego A, Keating C, Moodie M. Cost-effectiveness of whole-of-community obesity prevention programs: an overview of the evidence. *Expert Rev Pharmacoecon Outcomes Res*. 2014; 14: 719-27.
3 Karacabeyli D, Allender S, Pinkney S, Amed S. Evaluation of complex community-based childhood obesity prevention interventions. *Obes Rev*. 2018; 19: 1080-92.
4 Husereau D, Drummond M, Augustovski F, *et al.* Consolidated Health Economic Evaluation Reporting Standards 2022 (CHEERS 2022) statement: updated reporting guidance for health economic evaluations. *BMJ*. 2022; 376: e067975.
5 McAuley KA, Taylor RW, Farmer VL, *et al.* Economic evaluation of a community-based obesity prevention program in children: the APPLE project. *Obesity (Silver Spring)*. 2010; 18: 131-6.
6 Kuczmarski RJ, Ogden CL, Guo SS, et al.2000 CDC growth charts for the United States: methods and development. Data from the National Health Survey. *Vital Health Stat 11*. 2002; 246: 1-190.
7 Health Technology Analysts. Cost effectiveness report of public health interventions to prevent obesity. Report prepared for the Health Research Council of New Zealand: 2010.
8 Moodie ML, Herbert JK, de Silva-Sanigorski AM, *et al.* The cost-effectiveness of a successful community-based obesity prevention program: the be active eat well program. *Obesity (Silver Spring)*. 2013; 21: 2072-80.
9 Carter R, Moodie M, Markwick A, *et al.* Assessing cost-effectiveness in obesity (ACE-obesity): an overview of the ACE approach, economic methods and cost results. *BMC Public Health*. 2009; 9: 419.
10 Haby MM, Vos T, Carter R, *et al.* A new approach to assessing the health benefit from obesity interventions in children and adolescents: the assessing cost-effectiveness in obesity project. *Int J Obes*. 2006; 30: 1463-75.
11 Sanigorski AM, Bell AC, Kremer PJ, Cuttler R, Swinburn BA. Reducing unhealthy weight gain in children through community capacity-building: results of a quasi-experimental intervention program, Be Active Eat Well. *Int J Obes*. 2008; 32: 1060-7.
12 O'Connor DP, Lee RE, Mehta P, *et al.* Childhood Obesity Research Demonstration project: cross-site evaluation methods. *Child Obes*. 2015; 11: 92-103.
13 Foltz JL, Belay B, Dooyema CA, Williams N, Blanck HM. Childhood Obesity Research Demonstration (CORD): the cross-site overview and opportunities for interventions addressing obesity community-wide. *Child Obes*. 2015; 11: 4-10.
14 Ward DS, Benjamin SE, Ammerman AS, Ball SC, Neelon BH, Bangdiwala SI. Nutrition and physical activity in child care: results from an environmental intervention. *Am J Prev Med*. 2008; 35: 352-6.
15 Ananthapavan J, Nguyen PK, Bowe SJ, *et al.* Cost-effectiveness of community-based childhood obesity prevention interventions in Australia. *Int J Obes (Lond)*. 2019; 43: 1102-12.
16 Ananthapavan J, Sacks G, Brown V, *et al.* Priority-setting for obesity prevention-The Assessing Cost-Effectiveness of obesity prevention policies in Australia (ACE-Obesity Policy) study. *PLoS One*. 2020; 15: e0234804.
17 Brown V, Ananthapavan J, Veerman L, *et al.* The Potential Cost-Effectiveness and Equity Impacts of Restricting Television Advertising of Unhealthy Food and Beverages to Australian Children. *Nutrients*. 2018; 10.
18 Brown V, Moodie M, Cobiac L, Mantilla Herrera AM, Carter R. Obesity-related health impacts of fuel excise taxation- an evidence review and cost-effectiveness study. *BMC Public Health*. 2017; 17: 359.
19 Lal A, Mantilla-Herrera AM, Veerman L, *et al.* Modelled health benefits of a sugar-sweetened beverage tax across different socioeconomic groups in Australia: A cost-effectiveness and equity analysis. *PLoS Med*. 2017; 14: e1002326.

20 Waters E, Gibbs L, Tadic M, *et al.* Cluster randomised trial of a school-community child health promotion and obesity prevention intervention: findings from the evaluation of fun 'n healthy in Moreland! *BMC Public Health*. 2017; 18: 92.
21 de Onis M, Onyango AW, Borghi E, Siyam A, Nishida C, Siekmann J. Development of a WHO growth reference for school-aged children and adolescents. *Bull World Health Organ*. 2007; 85: 660-7.
22 Robinson TN. Reducing children's television viewing to prevent obesity: a randomized controlled trial. *JAMA*. 1999.

23 Rutishauser IHE, Webb K, Abraham B, Allsopp R. Evaluation of short dietary questions from the 1995 National Nutrition Survey. Canberrra. *Commonwealth Department of Health and Ageing*. 2001; 282: 561–7.
24 Adams JK, Huddy AD, Holden L, Newell SA, Miller M, Dietrich UC. Tooty Fruity Vegie – a recipe for successful volunteer participation in primary schools. *Health Promotion Journal of Australia*. 2003; 14: 187-91.
25 Huddy A, Adams JK, Holden L, Newell S, van Beurden E, Dietrich U. . Fruits and vegies in lunchboxes - accuracy of a prospective 24 hour food record for primary school children. *Health Promotion Journal of Australia*. 2003; 14: 141-3.
26 Ravens-Sieberer U, Gosch A, Rajmil L, et al. KIDSCREEN-52 quality-of-life measure for children and adolescents. Expert Rev Pharmacoecon Outcomes Res. 2005;5(3):353-364.
27 Bryant M, Burton W, Collinson M, *et al.* Cluster randomised controlled feasibility study of HENRY: a community-based intervention aimed at reducing obesity rates in preschool children. *Pilot Feasibility Stud*. 2018; 4: 118.
28 Gillespie J, Hughes A, Gibson AM, Haines J, Taveras E, Reilly JJ. Protocol for Healthy Habits Happy Homes (4H) Scotland: feasibility of a participatory approach to adaptation and implementation of a study aimed at early prevention of obesity. *BMJ Open*. 2019; 9: e028038.
29 Hughes AR, Stewart L, Chapple J, *et al.* Randomized, controlled trial of a best-practice individualized behavioral program for treatment of childhood overweight: Scottish Childhood Overweight Treatment Trial (SCOTT). *Pediatrics*. 2008; 121: e539-46.
30 Bell L, Ullah S, Leslie E, et al. OPAL evaluation project final report: OPAL phase 1 and 2. 2016.
31 Cole TJ, Flegal KM, Nicholls D, Jackson AA. Body mass index cut offs to define thinness in children and adolescents: international survey. *BMJ*. 2007; 335: 194.
32 Cole TJ, Bellizzi MC, Flegal KM, Dietz WH. Establishing a standard definition for child overweight and obesity worldwide:international survey. *BMJ*. 2000; 320: 1240-43.
33 Cole TJ, Freeman JV, Preece MA. Body mass index reference curves for the UK, 1990. Arch Dis Child. 1995;73(1):25-29.
34 Ratcliffe J, Couzner L, Flynn T, et al. Valuing Child Health Utility 9D health states with a young adolescent sample: a feasibility study to compare best-worst scaling discrete-choice experiment, standard gamble and time trade-off methods. Appl Health Econ Health Policy. 2011;9(1):15-27.
35 Ratcliffe J, Huynh E, Chen G, *et al.* Valuing the Child Health Utility 9D: Using profile case best worst scaling methods to develop a new adolescent specific scoring algorithm. *Soc Sci Med*. 2016; 157: 48-59.
36 Bryant M, Burton W, Cundill B, *et al.* Effectiveness of an implementation optimisation intervention aimed at increasing parent engagement in HENRY, a childhood obesity prevention programme - the Optimising Family Engagement in HENRY (OFTEN) trial: study protocol for a randomised controlled trial. *Trials*. 2017; 18: 40.
37 Swinburn B, Pryor J, McCabe M, *et al*. The Pacific OPIC project (Obesity Prevention in Communities)-objectives and designs. *Pac Health Dialog*. 2007; 14: 139-46.
38 Swinburn BA, Millar L, Utter J, *et al.* The Pacific Obesity Prevention in Communities project: project overview and methods. *Obes Rev*. 2011; 12 Suppl 2: 3-11.

39 Tran H, Killedar A, Tan EJ, *et al.* Cost-effectiveness of scaling up a whole-of-community intervention: The Romp & Chomp early childhood obesity prevention intervention. *Pediatric obesity*. 2022.
40 Haby MM, Doherty R, Welch N, Mason V. Community-based interventions for obesity prevention: lessons learned by Australian policy-makers. *BMC research notes*. 2012; 5: 20.
41 WHO Multicentre Growth Reference Study Group. WHO Child Growth Standards based on length/height, weight and age. *Acta paediatrica (Supplement)*. 2006; 450: 76.
42 Brown V, Tan E, Hayes A, Petrou S, Moodie ML. Utility values for childhood obesity interventions: a systematic review and meta-analysis of the evidence for use in economic evaluation. *Obesity reviews*. 2018; 19: 905-16.
43 StataCorp. Stata Statistic Software: Release 13. Texas: StataCorp LP: College Station 2015.
44 Brown V, Ananthapavan J, Sonntag D, Tan EJ, Hayes A, Moodie M. The potential for long-term cost-effectiveness of obesity prevention interventions in the early years of life. 2019; 14: e12517.
45 Reilly JJ, Methven E, McDowell ZC, *et al.* Health consequences of obesity. *Archives of disease in childhood*. 2003; 88: 748-52.
46 Hayes A, Tan EJ, Lung T, Brown V, Moodie M, Baur L. A New Model for Evaluation of Interventions to Prevent Obesity in Early Childhood. 2019; 10.
47 Tan EJ, Taylor RW, Taylor BJ, Brown V, Hayes AJ. Cost-Effectiveness of a Novel Sleep Intervention in Infancy to Prevent Overweight in Childhood. *Obesity*. 2020; n/a.
48 Coffield E, Nihiser A, Carlson S, *et al.* Shape Up Somerville's return on investment: Multi-group exposure generates net-benefits in a child obesity intervention. *Prev Med Rep*. 2019; 16: 100954.
49 Allender S, Millar L, Hovmand P, *et al.* Whole of Systems Trial of Prevention Strategies for Childhood Obesity: WHO STOPS Childhood Obesity. *Int J Environ Res Public Health*. 2016; 13.
50 Sweeney R, Moodie M, Nguyen P, *et al.* Protocol for an economic evaluation of WHO STOPS childhood obesity stepped-wedge cluster randomised controlled trial. *BMJ Open*. 2018; 8: e020551.
